# Enzyme Activity-Based Genome-wide Screening for Modifiers of Lysosomal Glucocerebrosidase Uncovers Candidate Risk Factors for Parkinson’s Disease

**DOI:** 10.1021/acscentsci.5c00240

**Published:** 2025-09-03

**Authors:** Vinod Udayar, Pierre-André Gilormini, Julien Bryois, Alexandra Gehrlein, Xi Chen, Stephanie Sonea, Sha Zhu, Matthew C. Deen, Nadia Anastasi, Alan E. Murphy, Nathan Skene, Manuela M. X. Tan, Jon-Anders Tunold, Filip Roudnicky, Wilma D. J. van de Berg, Lasse Pihlstrøm, David J. Vocadlo, Ravi Jagasia

**Affiliations:** ◧ Roche Pharma Research and Early Development, Neuroscience and Rare Disease Discovery and Translational Area, Roche Innovation Center Basel, 4070 Basel, Switzerland; ‡ Roche Pharma Research and Early Development, Therapeutic Modalities, Roche Innovation Center Basel, 4070 Basel, Switzerland; § Department of Molecular Biology and Biochemistry, 1763 Simon Fraser University, Burnaby, BC V5A 1S6, Canada; ∥ Department of Chemistry, 1763 Simon Fraser University, Burnaby, BC V5A 1S6 Canada; ⊥ UK Dementia Research Institute at Imperial College London, London SW7 2AZ United Kingdom; # Department of Brain Sciences, 4615 Imperial College London, London W12 0NN, United Kingdom; ○ Department of Neurology, 155272 Oslo University Hospital, Oslo 0424, Norway; □ Department of Anatomy and Neurosciences, Section Clinical Neuroanatomy and Biobanking, 1190 Vrije Universiteit Amsterdam, Amsterdam, 1081 HV Netherlands; △ Amsterdam Neuroscience, program Neurodegeneration, Amsterdam, 1081 HV Netherlands

## Abstract

Mutations in *GBA1*, the gene encoding the lysosomal hydrolase glucocerebrosidase (GCase), are the strongest common genetic risk factor for Parkinson’s Disease (PD). However, these mutations are incompletely penetrant, which suggests that there are likely genetic modifiers of GCase function. To identify such genes, we implemented a live cell GCase activity-based CRISPR-platform to enable genome-wide screening for novel regulators of lysosomal GCase activity. Among the screening hits, we find significant enrichment of genes linked to development and progression of PD through genome-wide association studies (GWAS). Moreover, we identify two lysosomal lipid transporter genes, including those encoding the lysosphospholipid transporter SPNS1 and the cholesterol transporter NPC1, and find an allele of SPNS1 that is associated with increased risk of PD. We show that disruption of SPNS1 does not affect GCase protein levels but impairs its lysosomal function. Collectively, these data suggest that dysfunction of many PD-associated genes converge to impact lysosomal GCase activity and thereby contribute to disease pathogenesis. A better understanding of the impacts of these and the other GCase modulators identified here should help unravel the important, yet complex, relationship between *GBA1* and PD.

## Graphical abstract

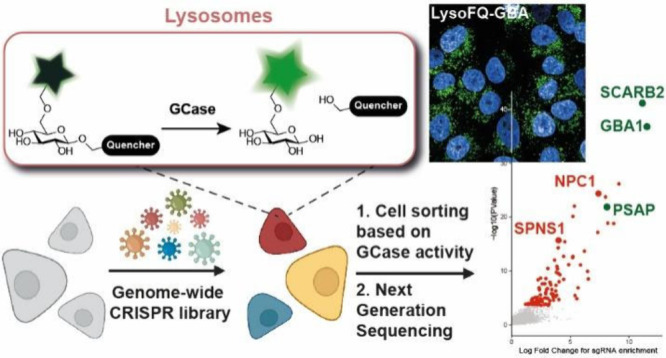

## Introduction

Proper functioning of lysosomes requires the activities of a large subset of proteins, and consequently, genetic alterations in many of these proteins result in lysosomal storage disorders (LSDs). Notable among this set of lysosomal proteins is the enzyme β-glucocerebrosidase (GCase), which is encoded by *GBA1*, and responsible for the hydrolysis of glucosylceramide (GlcCer). Loss of GCase enzymatic activity leads to lysosomal accumulation of GlcCer and its downstream metabolite, glucosylsphingosine (GlcSph). Biallelic mutations in *GBA1* causes Gaucher Disease (GD) and also place patients at a significantly increased risk of developing Parkinson’s Disease (PD).^1–3^ This observation led to the striking realization that even carrying one mutant allele of *GBA1* increases one’s risk of developing synucleinopathies, including PD, dementia with Lewy bodies (DLB), and rapid eye movement sleep behavior disorder (RBD).^4,5^ In addition, carriers of *GBA1* mutant alleles with PD not only have an earlier disease onset but also more rapid disease progression, suggesting that GCase serves a central role in the development of PD.^6^

While GCase has emerged as a key player in specific synucleinopathies, there is limited understanding of the factors modifying lysosomal GCase function. Some processes involved in the maturation of GCase have been defined. Trafficking of GCase to lysosomes depends on association in the endoplasmic reticulum (ER) with its obligate chaperone, the lysosomal integral membrane protein (LIMP)-2 (Figure 1a).^7^ LIMP-2 is itself modified by mannose-6-phosphate in the Golgi apparatus and is recognized by the mannose-6-phosphate (M6P) receptor (MPR), and the formation of a ternary complex of these proteins enables trafficking of GCase to lysosomes.^8^ However, it is important to note that an alternative proposal is that the LIMP-2-GCase complex traffics to lysosomes in an M6P-independent manner.^7^ Within lysosomes, GCase associates with saposin C, which enables the enzyme to exhibit its full catalytic activity.^9,10^ Disruption of the genes encoding these proteins leads to impaired GCase activity,^11^ which manifests in LSDs that resemble, in some respects, aspects of either GD or PD. These studies underscore that, beyond mutations within *GBA1*, the pathways influencing its activity could contribute to disease risk.

**Figure 1:**
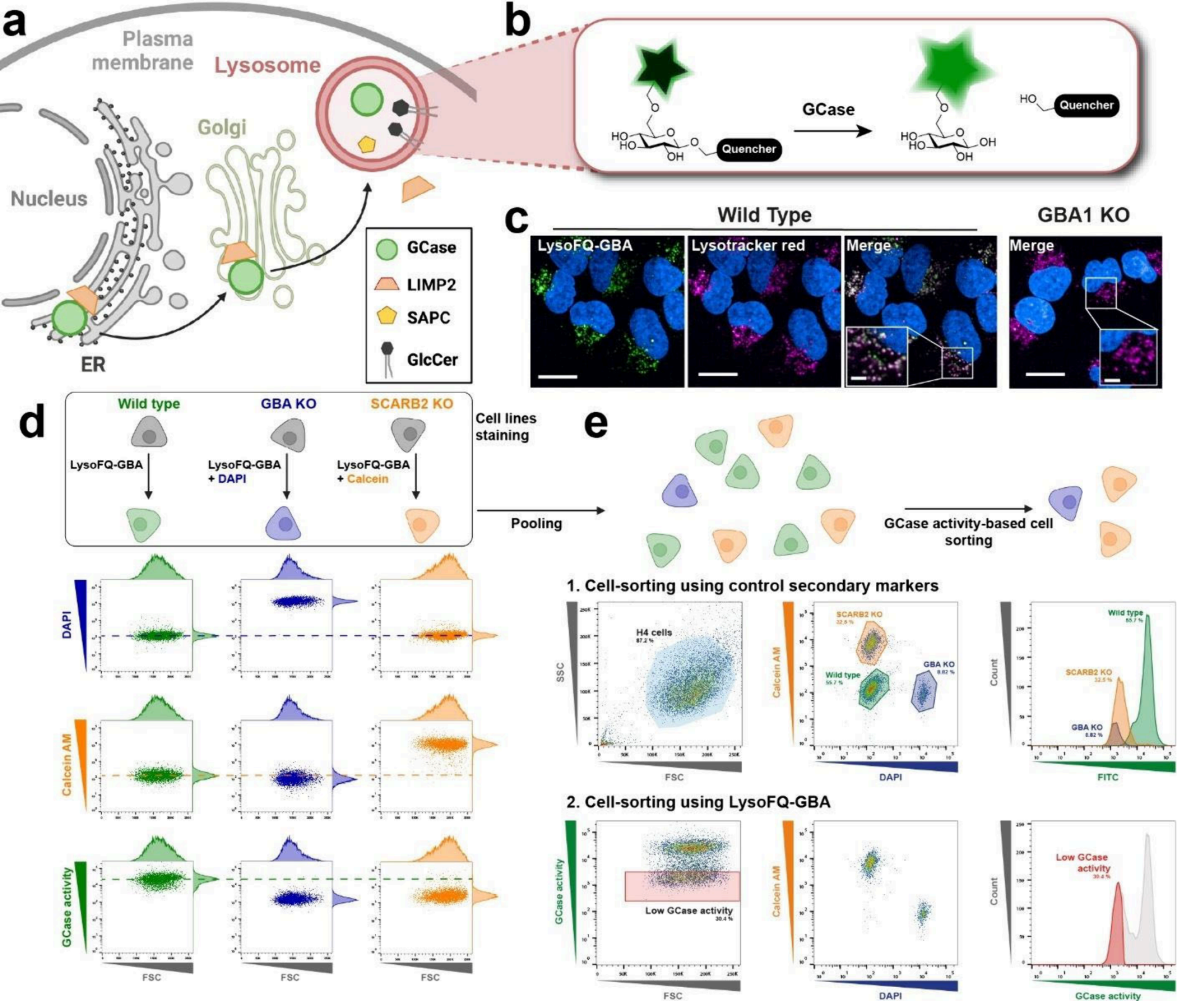
Development and validation of a fluorescence-assisted cell sorting strategy to isolate cells exhibiting low lysosomal GCase activity. (a) Schematic showing trafficking of GCase to lysosomes and the involvement of the well-known modifiers of GCase activity LIMP-2 and SapC. (b) Schematic showing the turnover of LysoFQ-GBA by GCase within lysosomes leads to a fluorescent product that is retained within lysosomes. (c) Confocal fluorescence imaging shows strong colocalization of the product of LysoFQ-*GBA* with SiR lyso and the near absence of LysoFQ-GBA turnover in *GBA1*-KO H4 cells. Scale bar overview: 100 μm; scale bar crop: 20 μm. (d) Strategy for marking low GCase activity *SCARB2*-KO H4 cells with Calcein (Orange) and *GBA1*-KO H4 cells with Hoechst (Blue) to illustrate the ability to distinguish low and high GCase activity cell populations by flow cytometry using LysoFQ-GBA. (e) Pooling of WT with marked SCARB2-KO H4 cells [Calcein (Orange)] and *GBA1*-KO H4 cells [Hoechst (Blue)], followed by incubation of the pool with LysoFQ-GBA, and cell sorting with gating on low GCase activity cells enables efficient separation of *SCARB2*-KO and *GBA1*-KO H4 cells from WT cells.

There has been progress in understanding the genes involved in the maturation and trafficking of GCase, yet the high clinical phenotypic variability and incomplete penetrance of *GBA1* mutations in GD remains unexplained. The source of this variability and limited penetrance, which is greater still in the context of haploinsufficiency and manifestation of PD,^12–15^ is consequently of interest and may arise from either or both genetic and environmental factors. Focusing on genetic factors, recent studies have proposed a small set of genes as modifiers of the penetrance of *GBA1* mutations, including leucine-rich repeat kinase 2 (*LRRK2*),^16–18^ Cathepsin B (*CTSB*),^13,19,20^ *SNCA*,^13,21^ and *TMEM175*.^13,22^ Impairment of some of these factors was found to diminish lysosomal GCase activity.^17,19,21^ The identification of these genes, coupled with wider genome-wide association studies (GWAS), has provided substantial genetic evidence that lysosomal mechanisms play a key role in PD pathogenesis. Yet, it is not clear whether these varied processes converge on lysosomal GCase activity.

To address this question and identify genes functioning as positive modulators of GCase activity, we developed a robust genome-wide CRISPR-Cas9 screening platform based on quantitative monitoring of lysosomal GCase activity. We leverage the GCase substrate LysoFQ-GBA, which is exquisitely selective for functional lysosomal GCase (Figure 1a).^23^ Applying LysoFQ-GBA to CRISPR-Cas9 screening, we identified genes known to regulate GCase, including *GBA1, SCARB2,* and *PSAP*, but we also uncovered many new positive regulators of GCase. Notably, these novel GCase regulators are significantly enriched in genes linked to PD through GWAS and also linked to disease progression. Finally, we examine the functional effects of three of these candidate modifiers and show how they regulate GCase activity in distinct ways. Collectively, these results reinforce the importance of *GBA1* and lysosomal mechanisms in Parkinson’s disease pathogenesis while also uncovering new genes that act upstream of *GBA1* to regulate lysosomal GCase activity.

## Results and Discussion

### Optimization of a Lysosomal GCase Activity-Based Assay in Live H4 Cells

To enable pooled CRISPR-Cas9 screening for modifiers of GCase activity, we set out to use the synthetic substrate LysoFQ-GBA to develop a flow cytometry-based assay to enable downstream fluorescence-assisted cell sorting of cell populations having differences in lysosomal GCase activity. The physicochemical properties of this synthetic substrate and its products enable convenient and reliable quantification of lysosomal GCase activity. Moreover, its dark-to-light switching (Figure 1b) make it well-suited to pooled cell-based screening by fluorescence-assisted cell sorting (FACS). As a model system, we chose neuroglioma cells, H4 cells since they have well-characterized GCase activity and are adequately robust to reliably survive cell sorting. We first evaluated the utility of LysoFQ-GBA in detecting lysosomal GCase in H4 cells by microscopy. This GCase assay involves the following steps: (i) seeding of cells and overnight culture, (ii) treatment of cells with the desired chemical or genetic perturbation, (iii) incubation with LysoFQ-GBA, (iv) termination of the GCase assay by removing substrate in the media and adding a highly selective GCase inhibitor, and finally (v) imaging of cells using confocal microscopy to quantify substrate turnover within lysosomes.

To validate suitability of this assay within H4 cells, we first confirmed LysoFQ-GBA was exclusively turned over by GCase (Figure 1c) by using H4 *GBA1* knockout (KO) cells that we generated using CRISPR-Cas9 mediated disruption of *GBA1*. We found that while consistent punctate fluorescence was observed in the wild type (WT) cells, no signal was observed in the *GBA1* KO cells. Next, to confirm LysoFQ-GBA is turned over within lysosomes, we analyzed the localization of fluorescent product using confocal microscopy with cells treated with both LysoFQ-GBA and SiR lysosomes as a lysosomal marker. These data showed a distinctive punctate pattern of fluorescence that colocalized with lysosomes (Figure 1c). Altogether, these initial data show that LysoFQ-GBA is cleaved selectively by only the lysosomal pool of GCase within H4 cells.

To pursue a suitably quantitative analysis of lysosomal GCase activity, we next performed dose- and time-response experiments with LysoFQ-GBA in order to determine conditions that could be reliably used to measure GCase activity within live cells (Figure S1). Our analyses revealed several regions of linear response for GCase catalyzed turnover of LysoFQ-GBA. From these regions we selected the dose and time falling within the largest linear range so as to obtain the largest dynamic range of signal to accurately measure changes in activity. Next, to confirm that LysoFQ-GBA can indeed quantitatively report on lysosomal GCase activity, we used well-known chemical inhibitors of GCase (AT3375 ^24^ and isofagomine^25^). Using these optimized conditions, we treated cells with a range of inhibitor concentrations and established the in-cell IC_50_ values (Figure S2) for these inhibitors acting on GCase as being ∼10 nM for AT3375 and ∼120 nM for isofagomine. The consistency of these values with the in vitro IC_50_ values (∼46 nM for AT3375 and ∼30 nM for isofagomine) observed for inhibition of GCase by these compounds, coupled with the observed Hill-slope of ∼1 (Figure S2), indicates that using these optimal assay conditions enables quantification of GCase activity using LysoFQ-GBA.

We next turned to validation of LysoFQ-GBA in flow cytometry-based assays by repeating the dose- and time-response experiments. Reassuringly, we found similar time and dose responsiveness using flow cytometry (Figure S3) as we had observed by microscopy. However, we consistently observed a greater standard deviation for all measurements irrespective of either the time or dose, suggesting that the increased variability in fluorescence intensity arises from the different modes of data collection and analysis (Figure S4). We conclude that cell-by-cell measurement using cytometry, as compared to integrated images of entire image fields, leads to this larger standard deviation in measurements due to inherent variability among individual cells within the overall cell population. We recognized that this variability has an impact on the resolution of the assay, a factor of paramount importance when one aims to sort cells based on their measured GCase activity. Accordingly, we set out to confirm the suitability of LysoFQ-GBA as a means of allowing the reliable identification and efficient separation of cells having different levels of GCase activity.

To assess the feasibility of a pooled CRISPR-Cas9 screen using flow cytometry, we devised a model experiment using three different H4 cell lines: WT, *GBA1* KO, and *SCARB2* KO. We reasoned that because *SCARB2* encodes the obligate GCase chaperone protein LIMP-2, its deletion should lead to a significant decrease, but not complete loss, of lysosomal GCase activity measured using LysoFQ-GBA. Thus, *GBA1* KO cells provided the maximum effect size for the assay, while *SCARB2* KO cells serve as a known pathway modifier of GCase activity. Using the optimized conditions, we treated each cell line with LysoFQ-GBA to measure its relative GCase activity. In addition, to track each cell population, we also treated the *GBA1* KO and *SCARB2* KO cells with readily distinguishable secondary fluorescent markers: Hoechst for staining nuclei of *GBA1* KO cells and Calcein Orange for staining the cytosol of *SCARB2* KO cells. We then separately analyzed each cell line by cytometry by measuring fluorescence in three channels: FITC (LysoFQ-GBA), DAPI (Hoechst), and TRITC (Calcein Orange; Figure 1d). These analyses confirmed we could reliably label H4 cells using these secondary markers. On analysis of GCase activity using LysoFQ-GBA, we observed markedly lower levels of lysosomal GCase activity in both *GBA1* KO (15-fold) and *SCARB2* KO (7-fold) cell lines. These assays confirm our results observed using fluorescence microscopy and the ability of LysoFQ-GBA to accurately report on lysosomal GCase activity by flow cytometry.

We next set out to confirm that we could reliably distinguish these populations of cells by sorting them from one another. To this end, we pooled the three cell lines together and then subjected the resulting mixture of cells to analysis by FACS. We then analyzed the cells using the secondary markers (Hoechst for *GBA1* KO cells, Calcein Orange for *SCARB2* KO, Figure 1e). When using two-dimensional analysis of Calcein Orange and Hoechst signals, the three different populations (WT [Hoechst^–^/Calcein^–^], GBA1 KO [Hoechst^+^/Calcein^–^], and SCARB2 KO [Hoechst^–^/Calcein^+^]) could easily be discriminated, quantified, and sorted. We then analyzed the same mixture using only GCase activity as a readout. This analysis revealed two distinct yet overlapping populations of cells having either low or high GCase activity (Figure 1e, panel 1). To examine whether we could isolate those cells having low GCase activity, we gated the population having lower GCase activity and then analyzed this population using the same two-dimensional analysis of Calcein Orange and Hoechst intensities. Strikingly, almost no WT cells [Hoechst^–^/Calcein^–^] were detected in this population, showing that both *GBA1* KO and *SCARB2* KO cells could efficiently be separated from the mixture using only GCase activity measured by LysoFQ-GBA. Further analysis showed that ∼91% of the *GBA1* KO and 62% of *SCARB2* KO cells could be retrieved from the mixture using only GCase activity as a readout (Figure 1e, panel 2). These combined experiments show that using LysoFQ-GBA not only allows one to measure changes in GCase activity upon perturbation but also enables the effective isolation of cells having low GCase activity from mixed populations of cells.

### Validation of a CRISPR-Cas9-Based Screening Strategy

Having established and verified a suitable protocol for sorting cells on the basis of their lysosomal GCase activity, we moved to implement this protocol to enable a pooled CRISPR-Cas9-based screening strategy. We initially focused on a set of 1496 genes known to be linked to lysosomal function, connected to PD through GWAS, or linked to levels of GCase protein in PD patient brain (Supporting Table S1). Notably, this set of genes included the positive controls affecting GCase activity that we used in the assay development (*GBA1*, *SCARB2*, and *PSAP*). We generated a library of lentiviral particles encoding eight distinct single guide RNA (sgRNA) molecules targeting each of these genes (Supporting Table S1). Using this library, we transduced a large population of H4 cells (HTB-148) that had been engineered to stably express Cas9 (H4-Cas9) (Figure 2a) at a multiplicity of infection (MOI) of 0.5 (Figure S5) and treated them with a puromycin to eliminate all nontransduced cells. In this way, we obtained a population of cells where a single gene is disrupted within each cell. We then expanded this cell population and treated it with LysoFQ-GBA. We also added to this sample, as a reference control, a small number of WT H4 cells (nontransduced) treated with both LysoFQ-GBA and Hoechst. The GCase activity of these DAPI-positive WT H4 cells was used to accurately gate (Figure 1e; Figure S6a) the population of cells defined as having a lower GCase activity, which were then collected by cell sorting. Half of these enriched H4 cells were preserved for later sequencing. The other half were cultured and sequentially used for a second round of enrichment using treatment with LysoFQ-GBA with half of the cells at each step being stored for downstream sequencing analyses. We observed that in the second round of enrichment a larger fraction of the H4 cell population exhibited lower GCase activity, indicating that our gating strategy enabled isolation of cells in which a disrupted gene resulted in lower GCase activity (Figure S6b). Nevertheless, we found that only one round of enrichment was adequate and does not change the set of genes identified. Ultimately, we used the data and fold-changes from round 2 for detailed analyses. Finally, we extracted the genomic DNA from the cells preserved from each round that had low GCase activity, as well as nontransduced cells for baseline reference, and processed these samples for Next Generation Sequencing (NGS).

**Figure 2:**
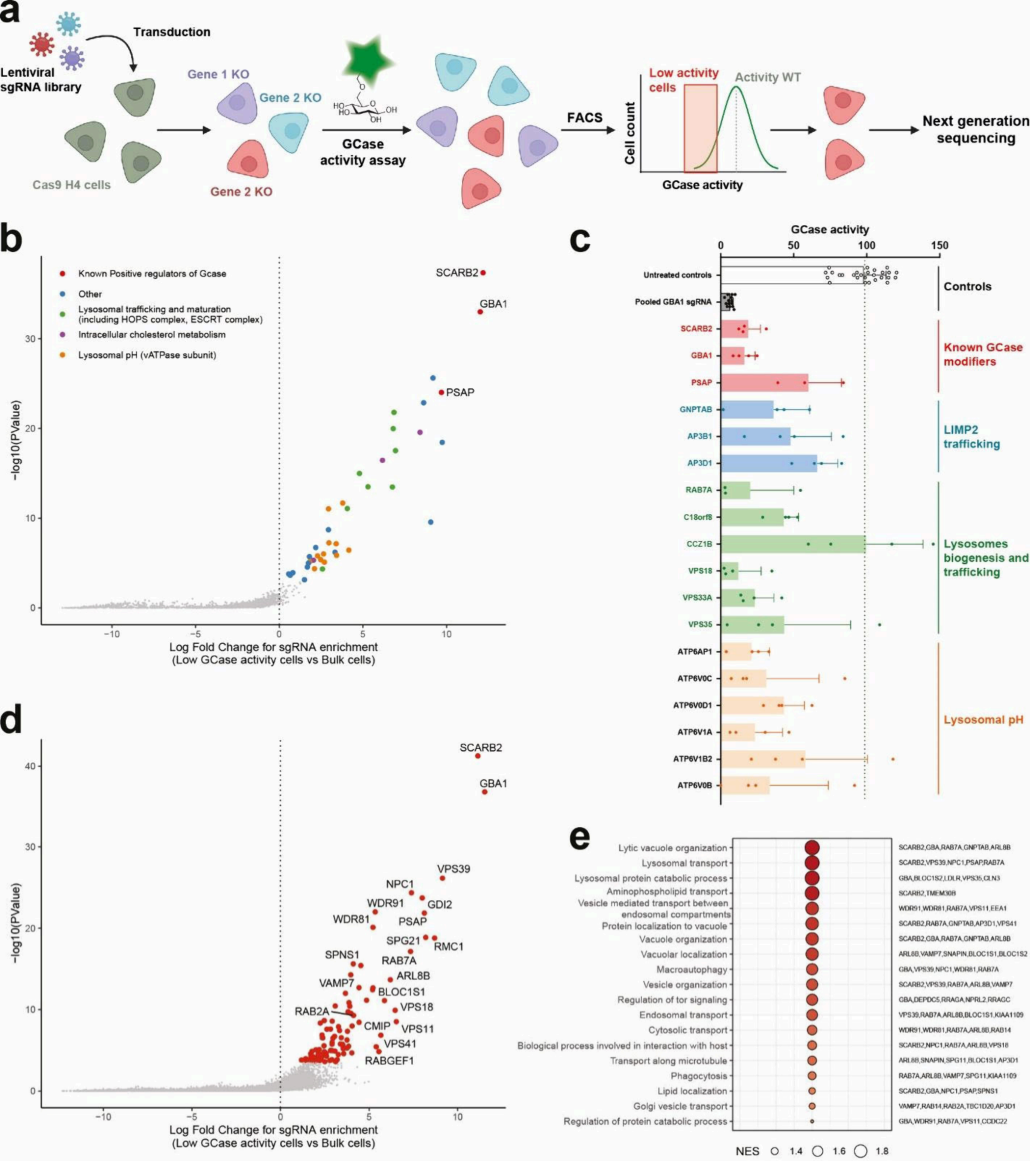
LysoFQ-GBA reveals hits from both targeted and genome-wide CRISPR-Cas9 screening. (a) Schematic workflow of an activity-based pooled CRISPR-Cas9 screen with Gcase activity as the sorting parameter. Three rounds of enrichment are included in the workflow. (b) Results of the targeted CRISPR-Cas9 pooled screen and ranking of the most significant hits. (c) Validation of the most significant hits from the targeted screen in an arrayed CRISPR-Cas9 activity-based assay. Each data point represents a different sgRNA targeting the corresponding gene. Error bars show standard deviation (SD). (d) Results of the genome-wide CRISPR-Cas9 pooled screen and ranking of the hits. (e) Pathway analysis showing those pathways most frequently observed among the ranked genes.

Using the resulting sequencing data, we identified those sgRNA that led to the disruption of their target genes within these cell populations. We then determined the extent of enrichment of sgRNA for each gene compared to the nontransduced cells (Log Fold Change, LogFC) (Supporting Table S1) and, using this approach, we identified 40 genes (Figure 2b, Supporting Table S2) for which disruption significantly impaired GCase activity (False Discovery Rate (FDR) < 5%, p-value <10^–5^). As expected, both *SCARB2* and *GBA1* were the most highly enriched genes. Reassuringly, we also identified *PSAP*, the gene encoding prosaposin, which is essential for the lysosomal activity of GCase.^26^ In addition, a number of genes were identified that made mechanistic sense including *GNPTAB*, which encodes *N*-acetylglucosamine-1-phosphotransferase, the enzyme responsible for installation of the M6P moiety that directs the GCase-LIMP-2 complex to lysosomes.^7^ Notably, we also observed *AP3B1* and *AP3D1*, which are both linked to Hermansky-Pudlak Syndrome^27^ and encode facilitators of vesicular budding from the Golgi membrane that are known to regulate LIMP-2 trafficking. These data illustrate the ability of this screen to identify genes influencing the GCase activity through multiple degrees of separation. From among our observations (Supporting Table S2) there are several that are directly linked to lysosomal homeostasis. Indeed, we also observed 12 genes encoding subunits of the Vacuolar-type ATPase (V-ATPase) (*ATP6 V0D1*, *ARP6 V1C1*, *ATP6 V1H*, *ATP6 V1D*, *ATP6 V1G1*, *ATP6AP1*, *ATP6 V0C*, *ATP6 V1F*, *ATP6 V1A*, *ATP6 V1E1*, *ATP6 V0B*, *ATP6 V1B2*), of which *ATP6 V0C*, *ATP6 V1A*, and *ATP6 V0A1* are linked to neurodevelopmental disorders,^28^ underscoring the importance of the V-ATPase in maintaining the acidic environment of lysosomes coupled with the known pH-sensitivity of GCase.^29^ We also observed the enrichment of several other families of genes linked to trafficking.

These included a group of eight genes related to one of the endosomal sorting complexes required for transport (ESCRT II, *VPS36*)^30^ as well as the homotypic fusion and protein sorting (HOPS)-tethering and Class C core vacuole/endosome tethering (CORVET) complexes (*VPS39, VPS33A*, *VPS18*, *VPS41*, *VPS16*, *VPS11*).^31^ We also noted the involvement of *Rab7A* and *Rab2A*, which are members of the Rab family of GTPases, which influence lysosomal biogenesis and membrane fusion—as well as genes encoding activators of Rab7A: *C18orf*8 and *CCZ1b*.^32^ Consistent with these observations pertaining to enrichment of trafficking proteins, we also identified *VPS35*, a gene encoding the vacuolar protein sorting ortholog 35 which is involved in autophagy and has been genetically linked to neurodegenerative diseases including PD.^33^ In summary, these data show high enrichment of genes that are already well-known to influence GCase activity (*SCARB2*, *PSAP*, and *GBA1*) as well as many genes that one would reasonably expect to influence lysosomal GCase activity by virtue of their influence on lysosomal maturation and pH. We therefore concluded that this live cell enzymatic assay enables identification of both direct and indirect GCase modifiers of GCase activity.

To validate these candidate genes, we performed this activity assay in an array-based format to achieve greater precision by using microscopy, which, as noted above, enables greater signal averaging. For each of the 40 genes, we selected the four most efficient sgRNA based on their fold enrichment (Supporting Table S2) and assembled these in separate wells for each gene in a 96-well microplate format. After the cells were seeded, transduced, and cultured, we treated each well with LysoFQ-GBA and then quantified GCase activity. *GBA1*, *SCARB2*, and *PSAP* KO cells all showed a clear reduction in GCase activity (Figure 2c). The majority (37 of 40) of disrupted genes observed in the primary screen were replicated in this secondary screen (Supporting Table S3). Notably, however, the significance of the enrichment of a gene does not necessarily correlate with the effect size on the lysosomal GCase activity. We reasoned this may stem from loss of some genes influencing the rate of cell growth and therefore skewing enrichment in the resulting sorted population. Another possible contributing factor is differences in the effectiveness of each sgRNA. Nevertheless, the extent by which two genes linked to the same biological process reduce GCase activity might reflect the relative importance of this gene in the underlying biological process. For example, *ATP6 V1B2* (Relative GCase activity = 58%) might have a lower impact on lysosomal pH regulation than its counterpart *ATP6 V1A* (Relative GCase activity = 24%). In any event, this array-based confirmatory analysis using microscopy-based analyses validates the efficiency of the FACS strategy and downstream data analysis used to identify genes influencing lysosomal GCase activity.

### Genome-wide Pooled CRISPR Screen

In light of the promising results obtained in our pilot screen, we decided to expand our screening to the genome-wide level. The chosen library contains 72726 sgRNAs, targeting 18485 genes (Supporting Table S4). We applied our optimized procedures but used a greater number of cells to enable the increased number of sgRNA used in this screen. After three rounds of serial enrichment, each done with three independent cell populations, the resulting DNA samples were sequenced. The results were processed using the same criteria as used for the pilot screen (Figure 2d, Supporting Table S4). When comparing the different rounds of enrichment with the results of the pilot screen, we found them to be comparable (Figure S7), confirming that only one round of enrichment was adequate to identify significantly enriched genes. Ultimately, we used the data and fold-change from round 3 for detailed analyses. Most of the highly enriched genes from the pilot screen were also observed in the genome-wide screen including *SCARB2* (logFC = 11.14, p-value = 5.35 × 10^–42^) and *GBA1* (logFC 11.53, p-value = 1.74 × 10^–37^), with *PSAP* (logFC = 8.12 and p-value = 3.70 × 10^–19^) following closely behind. In this regard, we found a strong correlation in the logFC (*R* ^2^ = 0.71, p-value of 4.9 × 10^–7^) between hits observed in the pilot screen and those in the genome-wide screen (Figure S8). Together, these data show the high reproducibility of our screen and confirmed successful implementation of the assay at the genome-wide level.

Each of the main classes of genes identified in the pilot screen were verified in the genome-wide screen and augmented by the presence of additional genes having related functions. Indeed, many genes related to lysosomal biogenesis and trafficking were identified. For example, further members of the Rab family of GTPases were highly ranked (*RAB14*, *RAB2A*) as well as several genes known to facilitate or activate Rab activity (*GDI2*, WDR91, PDZD8, *TBC1D20*, and *DENND6A*). Also, additional genes involved in the HOPS complex (*ARL8B*), the fusion of vesicles membrane (*VAMP7*, *BLOC1S2, SNAPIN*), and trafficking through the secretory pathway (*TRAM2, SERP1, LYST*) were identified. These results confirm that GCase activity is strongly dependent on the appropriate lysosomal biogenesis. Perhaps more interesting was that disruption of several genes related to neurologic diseases such as spastic paraplegia (*SPG21*, *SPG11*, *ZFYVE26*, *ZFYVE27*, *AP5S1*, *SELENOI*), epilepsy (*DEPDC5*), nephrotic syndrome (*CMIP*), ataxia (*GDAP2*), and PD (*SCARB2*, *GBA1*, *SPTSSB*, *RABGEF1*, *ARL8B*, *GNPTAB*, *RAB14*, *NPC1*, *VPS39*, and others) all significantly reduced GCase lysosomal activity.

### Genetic Risk Factors for Parkinson’s Disease Are Enriched among Genes Impacting GCase Activity

Notably, we found that the hits identified from our genome-wide screen were enriched in genetic associations with Parkinson’s disease using data from a recent^34^ PD GWAS (P-value = 0.00056) (Figure 3a,b), suggesting that PD genetics may in part converge on GCase activity. We note that this result was specific to PD as we did not observe genetic enrichment for Alzheimer’s disease and schizophrenia (Figure S9). Several of the genes we identified as influencing GCase activity were linked in various ways to lipid metabolism, including, for example, *SELENOI*, *SPNS1*, *LDLR*, and *NPC1*. Since *NPC1* and *SPNS1* strongly affected both GCase activity and are both lysosomal transporters of lipids/cholesterols, we selected these genes for further study. Intriguingly, *SPNS1* is situated near an established GWAS locus associated with PD (Figure 3c),^35^ suggesting that this GWAS signal may reflect a genetic variant influencing SPNS1 function. To investigate this possibility, we performed a genetic colocalization analysis using Coloc^36^ to determine if the GWAS locus and expression quantitative trait loci (eQTL) for *SPNS1* captured the same genetic signal. Our analysis yielded a high posterior probability of a shared genetic signal: 97% and 92% for the Lopes^37^ and Kosoy^38^ data sets respectively, two eQTL data sets mapping effects of single nucleotide polymorphism (SNP) on gene expression in microglia (Figure 3c). Mendelian randomization provided significant evidence for a causal relationship between *SPNS1* expression and PD risk; beta_smr = −0.3/–0.18, p-value = 6.3 × 10^–4^/0.0017 for the Lopes and Kosoy data sets, respectively. Also consistent with our observation that *SPNS1* disruption led to reduced GCase activity, we observed that the allele associated with decreased SPNS1 expression was associated with an increase in PD risk, perhaps by reducing the GCase function. Collectively, these data from our CRISPR screen and human genetic analyses robustly nominate *SPNS1* as a genetic determinant of PD risk, likely through its modifying effect on GCase activity. Given that the known PD risk factor *SCARB2* ^35,39^ also appears with high probability in our data set, we expect that other genes identified in our CRISPR screen are likely modifiers of lysosomal GCase activity and speculate that mutant alleles in these genes may eventually emerge as risk factors for PD.

**Figure 3:**
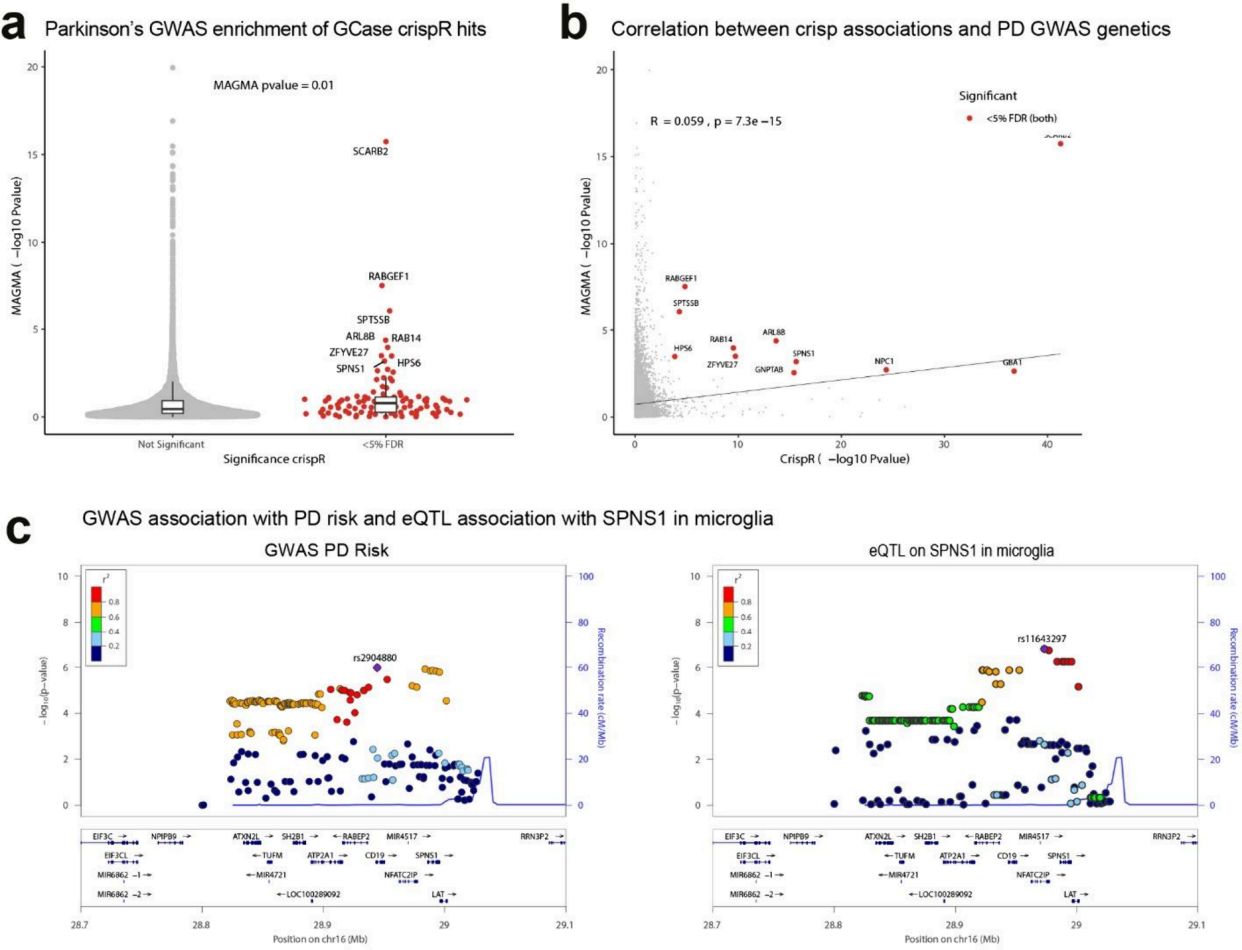
Human genetic evidence linking GCase regulators and Parkinson’s disease risk. (a) Parkinson’s disease GWAS genetic enrichment of significant GCase CRISPR screen hits (<5% FDR). (b) Correlation between the strength of evidence for our GCase CRISPR screen hits (−log_10_P-value) and the gene-level PD GWAS strength of evidence (−log_10_P-value). (c) Locus plots showing the colocalization of the PD GWAS association (left) and SNPs affecting *SPNS1* expression in microglia.^37^

### An Improved Lysosomal Polygenic Risk Score Is Associated with Both Parkinson’s Disease Risk and Progression to Dementia

We recently showed that lysosomal polygenic burden is associated with higher Lewy body Braak stage^40^ and faster progression to dementia^41^ in the subset of patients that have low AD copathology or AD polygenic risk, respectively. Since several hits including, for example, *SCARB2* are part of the PRS and the CRISPR screen links additional genes to lysosomal dysfunction through reduced GCase activity, we hypothesized that a revised lysosomal PRS incorporating these novel candidate genes would show an even stronger association with the same neuropathological and clinical outcomes. At the genome-wide significance threshold of *p* < 5 × 10^–8^, the revised lysosomal PRS included two additional loci that have not been previously included, which are proximal to the *SPNS1* and *SPTSSB* genes. In data from Netherlands Brain Bank donors, we analyzed data from the subset of patients with low AD copathology in an ordinal regression model with Lewy body Braak stage as the outcome. The revised lysosomal PRS incorporating CRISPR screen nominated genes showed a higher OR and lower p-value than the standard lysosomal PRS (Table 1). We then assessed time to dementia in data from the Parkinson’s Progression Markers Initiative (PPMI) using cox regression in the subset of patients with low AD polygenic risk and found that the hazard ratio (HR) of the lysosomal PRS increased from 1.89 to 1.99 as *SPNS1* and *SPTSSB* loci were incorporated in the score. For a similar analysis in the Parkinson’s Disease Biomarker Program (PDBP) cohort, the HR was unchanged. Taken together, these results lend support to the hypotheses that genetic variation near *SPNS1* and *SPTSSB* contributes to lysosomal polygenic burden and may be implicated in the neuropathological and cognitive progression of PD.

**Table 1:** Inclusion of *SPNS1* and *SPTSSB* from CRISPR Screening Provides a Revised Lysosomal PRS with Stronger Association with Parkinson’s Disease Progression

**Polygenic Risk Burden (PRS)**	**OR/HR (95% CI)**	**P-value**
***Lewy pathology ordinal regression - Netherlands Brain Bank (low AD-co pathology subset)***		
Lysosomal PRS	1.48 (1.04–2.09)	0.027
Lysosomal + GCase screen PRS	1.54 (1.09–2.18)	0.016
***Progression to dementia, cox regression - PPMI (low AD-PRS subset)***		
Lysosomal PRS	1.89 (1.24–2.88)	0.0032
Lysosomal + GCase screen PRS	1.99 (1.32–3.01)	0.0011
***Progression to dementia, cox regression–PDBP (low AD-PRS subset)***		
Lysoosmal PRS	1.31 (1.08–1.58)	0.0054
Lysosomal + GCase screen PRS	1.31 (1,07–1.59)	0.0074

### Characterization of Cellular Phenotype Associated with Disruption of *NPC1*, *SPNS1*, and *SCARB2*

Based on our screening results in combination with the genomic analysis implicating both *NPC1* and *SPNS1* in PD, we decided to perform more targeted cellular studies to understand the effects associated with the disruption of these genes. *SPNS1* was recently identified as a lysophospholipid transporter, responsible for the lysosomal efflux of lysophosphatidylcholine (LPC) and lysophosphatidylethanolamine (LPE).^42,43^

Interestingly, *SELENOI*, which was also observed as a highly ranked gene within the screen, encodes a transmembrane protein responsible for the biosynthesis of phosphatidylethanolamine, reinforcing the importance of phospholipid metabolism for GCase activity and lending further support for involvement of *SPNS1* function influencing lysosomal GCase activity. We were also drawn to *NPC1*, which encodes a transporter responsible for the recycling of cholesterol and which also appears to facilitate transport of sphingosine out of the lysosomes.^44^ Disruption of *NPC1* leads to accumulation of lipid products within lysosomes, causing Niemann-Pick disease, a rare lysosomal storage disorder (LSD).^45^ Studies have not found evidence for *NPC1* being linked to PD;^46,47^ however, this could stem from a lack of statistical power. Indeed, in the PD GWAS we analyzed, we found significant associations between SNPs around *NPC1* and PD risk (MAGMA FDR < 5%) (Figure 3b). Furthermore, we noted that disruption of either *SPNS1* or *NPC1* leads to a cellular phenotype in which multilamellar features can be detected within lysosomes by electron microscopy.^48,49^ Significantly, such a cellular phenotype is also seen in cells having either one or two loss-of-function alleles of *GBA1*, including cells from PD patients harboring *GBA1* mutations.^50^ Though defective SPNS1 transporter has not been linked in humans to a specific LSD, based on these noted phenotypic similarities in combination with the marked effect of *SPNS1* and *NPC1* disruption on GCase lysosomal activity, we set out to examine the mechanism by which impaired functioning of *SPNS1* and *NPC1* might influence GCase activity. In parallel, we also decided to compare the effects arising from loss of *SPNS1* or *NPC1* function with those arising from loss of LIMP2, which is well-known to impair trafficking of GCase to lysosomes.^7^

To explore how disruption of *SPNS1* and *NPC1* affect lysosomal GCase activity, we generated monoclonal knockouts of *SPNS1* and *NPC1* in H4 cells (H4 *NPC1* KO, H4 *SPNS1* KO, Figures 4 and S10). To compare the effects arising from loss of these two genes with loss of a gene linked to GCase function in a well-known manner, we also used a polyclonal *SCARB2* knockout population of H4 cells (H4 *SCARB2* KO) since *SCARB2* was also the highest ranked gene in our screen. Using these cells, we first set out to assess whether *SPNS1* and *NPC1* disruption resulted in a broader effect on lysosomal function beyond reducing GCase activity. We therefore examined H4 *NPC1* KO and H4 *SPNS1* KO cells using four different probes (Figure 4a,b,c): LysoFQ-GBA substrate (lysosomal GCase activity), GalBABS substrate (lysosomal α-galactosidase A [GalA] activity),^51^ Magic Red (Cathepsin B activity), and Lysotracker green (lysosomotropic reporter). As expected from the screening results, knockout of *GBA1*, *SCARB2*, *NPC1*, and *SPNS1* each led to cells exhibiting reduced lysosomal GCase activity. However, using GalBABS we found that lysosomal GalA activity was elevated (3–4 fold) in both the *NPC1* and *SPNS1* KO cells as compared to both the WT and the *GBA1* KO cells (Figure 4a,b and Figure S11). Lysotracker green signal was also drastically higher in all three KO cells lines, although to varying extents (15–20 fold for *SPNS1*, 5 fold for *NPC1*, 2.5 fold for *GBA1*). Finally, compared to the WT cells, Cathepsin B activity showed a significant decrease in *SPNS1* KO cells, a small but statistically significant increase in *NPC1* KO and in *SCARB2* KO (Figure 4a,c), and no change in the *GBA1* KO cells. Altogether, these data support the idea that the genetic disruption of *SPNS1* and *NPC1* has distinctive effects on the lysosomal environment that extend beyond the simple impairment of GCase activity.

**Figure 4:**
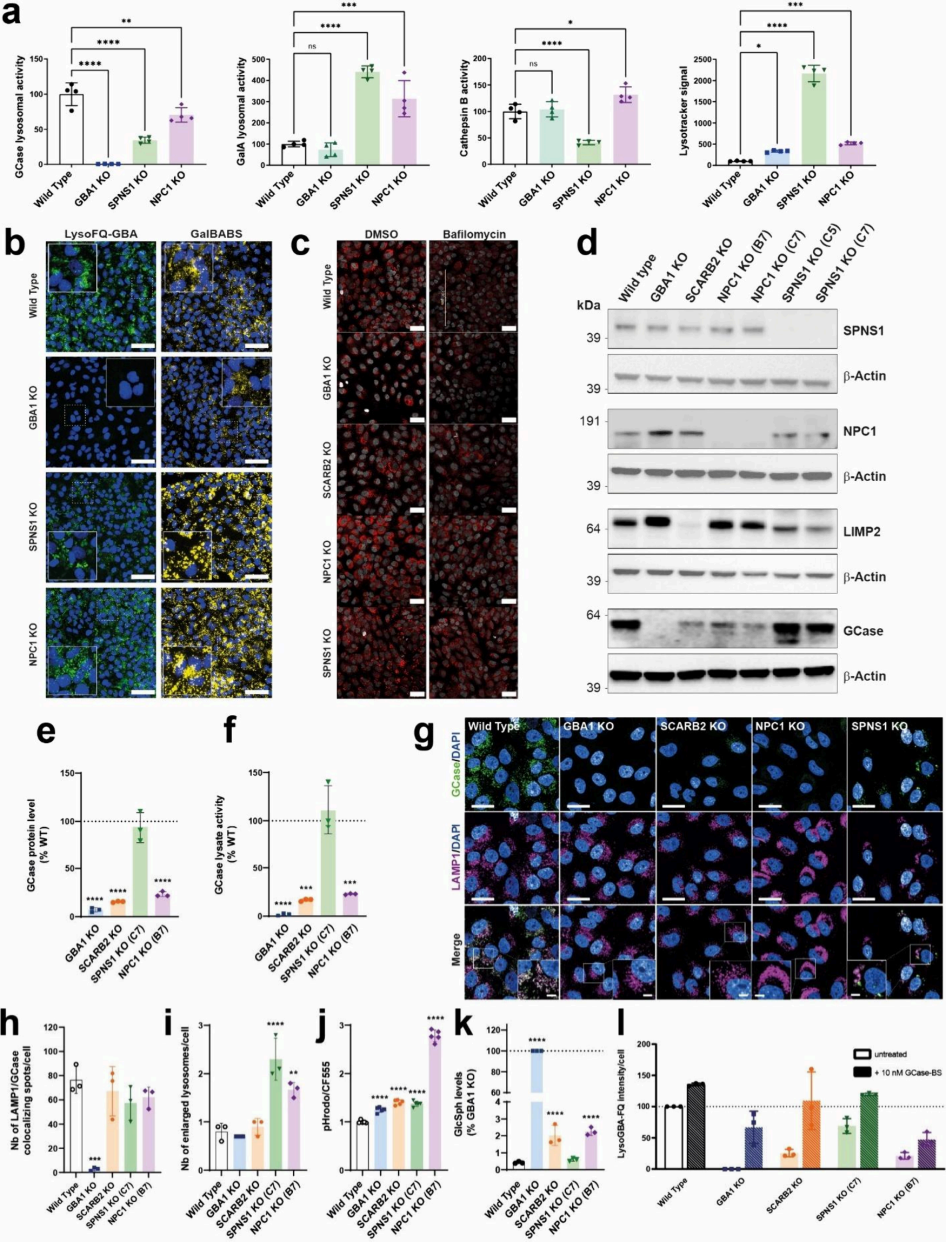
*SCARB2*, *NPC1*, and *SPNS1* regulate lysosomal GCase activity in distinct ways. (a) Quantitative activity measurements of various lysosomal enzymes–GCase, αGalA, Cathepsin B–and lysotracker staining in Wild Type, *GBA1* KO, *NPC1* KO, and *SPNS1* KO H4 cell lines. (b) Representative images of an activity-based assay targeting GCase (LysoFQ-GBA) and αGalA (GalBABS) in H4 cell lines. Scale bar: 50 μm. (c) Representative images of activity-based assay targeting Cathepsin B (Magic Red substrate) with or without treatment with Bafilomycin A (10 nM). Scale bar: 50 μm. (d) Immunoblots of SPNS1, NPC1, LIMP2 and GCase, in various H4 cell lines. (e) Quantification of the GCase level by immunoblot in various H4 cell lines. (f) GCase lysate activity measurements in, *GBA1* KO, *SCARB2* KO, *SPNS1* KO, and *NPC1* KO H4 cells. (g) Representative micrographs after immunostaining of GCase and LAMP2 in Wild Type, *GBA1* KO, *SCARB2* KO, *NPC1* KO and *SPNS1* KO H4 cells. Scale bar overview: 100 μm; scale bar crop: 20 μm. (h) Colocalization between GCase and LAMP1 in Wild Type, *GBA1* KO, *SCARB2* KO, *NPC1* KO and *SPNS1* KO H4 cells. (i) Number of enlarged lysosomes in Wild Type, *GBA1* KO, *SCARB2* KO, *NPC1* KO, and *SPNS1* KO H4 cells. (j) Relative lysosomal pH in Wild Type, *GBA1* KO, *SCARB2* KO, *NPC1* KO, and *SPNS1* KO H4 cells. (k) Glycolipids levels in WT, *GBA1* KO, *NPC1* KO, and *SPNS1* KO H4 cells. (l) GCase activity in Wild Type, *GBA1* KO, *SCARB2* KO, *NPC1* KO, and *SPNS1* KO H4 cells with or without prior-treatment with 10 nM GCase Brainshuttle. In panels a, e, f, h, j, k, and l, each data point represents measurement of an independent experimental replicate. Statistical significance was assessed using a one-way ANOVA, test. *P* ≤ 0.05 (*), *P* ≤ 0.01 (**), *P* ≤ 0.001 (***), and *P* ≤ 0.0001 (****).

Based on these considerations, we reasoned that disruption of these various genes may act either directly on GCase in a rapid manner or, more slowly, in an indirect manner by altering the lysosomal environment. We therefore monitored the change in lysosomal GCase activity over time following transfection of Cas9-expressing H4 cells with sgRNAs targeting *GBA1*, *SCARB2*, *PSAP*, *SPNS1*, and *NPC1* (Figure S12). As expected, *GBA1* disruption resulted in a rapid reduction of lysosomal GCase activity, observed after just 1 day. Disruption of *SCARB2* and *PSAP* showed an effect after 2 or 3 days. However, impaired GCase activity upon disruption of *NPC1* and *SPNS1* was only observed later, after 6–8 days. These results suggest that loss of function of these lipid transporters likely affects GCase activity in an indirect manner through time-dependent changes that gradually arise within the cellular environment.

We next set out to examine more specifically how these genetic perturbations might influence lysosomal GCase activity and to contrast these effects against those seen for the loss of *GBA1* and *SCARB2*. Immunoblot analyses confirmed the disruption of the target genes and their corresponding proteins (Figure 4d,e). Analysis of GCase protein levels revealed an expected decrease within *SCARB2* KO cells, as well as *NPC1* KO cells (25–50%), whereas GCase levels remained unaffected within *SPNS1* KO cells (Figure 4d,e). We next analyzed the overall levels of GCase activity within cells by measuring GCase activity in cell lysates using resorufin β-d-glucopyranoside (Res-Glc; Figure 4f). Values from this assay reflect the activity of the entire cellular pool of GCase and showed results that were consistent with a decrease of lysosomal GCase activity in the H4 *NPC1*, *GBA1*, and *SCARB2* KO cells relative to WT cells. However, we observed no significant difference in GCase activity within lysates from H4 *SPNS1* KO cells as compared with WT cells. These data suggest that the loss of *SPNS1* likely impairs the maturation or trafficking of GCase to lysosomes but not its overall levels.

Since the changes in GCase protein levels (Figure 4e) and overall cellular GCase activity (Figure 4f) cannot explain the differences we observed in decreased lysosomal GCase activity arising from disruption of *SPNS1* (Figure 4a,b), we went on to perform immunocytochemical analyses using an antibody^52^ that detects folded GCase. Examining WT, *GBA1*, *SCARB2*, *NPC1*, and *SPNS1* KO cell lines, using LAMP1 as a lysosomal marker, when we accounted for the decreased levels of folded GCase seen in the knockout lines, we observed a trend toward a lower extent of colocalization between GCase and LAMP1 in the *GBA1*, *SCARB2*, and *NPC1* cell lines (Figure 4g,h), which was generally consistent with our immunoblot results (Figure 4d,e). In the *SPNS1* KO cells, however, we observed a decrease in the amount of mature folded lysosomal GCase (Figure 4h), but immunoblot data showed no major change in overall GCase protein levels (Figure 4d,e). A LAMP1-based analysis of lysosomal morphology of *SPNS1*, *NPC1*, *SCARB2*, and *GBA1* KO lines as compared to WT cells revealed that, while *GBA1* and *SCARB2* KO cells showed no lysosomal enlargement, disruption of *NPC1* and *SPNS1* led to significantly enlarged lysosomes (Figure 4i, Figure S13), consistent with previous observations^48,49^ that disruption of *NPC1* and *SPNS1* leads to abnormal lysosomal morphology. The observation that disruption of *SPNS1* and *NPC1* leads to effects not seen in *GBA1* KO cells suggests that these two lysosomal transporters act upstream of GCase, in different ways, to alter the lysosomal environment. Further, these data suggest that although overall GCase protein levels are unaffected by disruption of *SPNS1*, lysosomal levels of folded GCase are decreased.

To further explore how the disruption of *SPNS1* and *NPC1* alters the lysosomal environment, we next examined the lysosomal pH and cellular levels of free cholesterol within these KO cell lines. We were motivated to examine lysosomal pH by previous reports suggesting a link between *SPNS1* and the regulation of lysosomal acidity.^53^ Levels of cholesterol were interesting because loss of *NPC1* function is known to increase cellular cholesterol and an increase of serum cholesterol has been reported in *SPNS1* knock-down mice.^42^ To estimate the luminal lysosomal pH, cells were treated with pH-sensitive and pH-insensitive dextrans conjugated with different fluorophores. The fluorescence ratio of these two dyes was used to compare the relative lysosomal acidity of our set of cell lines (Figure 4j). Loss of *GBA1*, *SCARB2*, and *SPNS1* all appeared to cause a small but statistically significant acidification of lysosomes, whereas loss of *NPC1* resulted in more pronounced acidification. These findings do not support the hypothesis that *SPNS1* disruption is linked to the dysfunction of the V-ATPase pump.

To assess changes in the amount of free cholesterol within cells, we stained the set of cell lines using filipin, a fluorescent polyene antibiotic that binds to cholesterol (Figure S14). A moderate decrease in staining intensity was observed in the *GBA1* KO cells compared to WT cells, but the *NPC1* KO cells showed the expected increase in fluorescence associated with accumulation of cellular cholesterol. Notably, *SPNS1* KO cells also showed an increase in the level of filipin staining. Image analysis showed the number of filipin positive vesicles decreased in both *SPNS1* and *NPC1* KO cells but not in *GBA1* KO cells, while the size of such vesicles increased. Next, to assess the consequences of disrupting these genes on the activity of lysosomal GCase function toward its endogenous substrates, we examined the levels of cellular GlcSph, one of the known substrates of GCase, and found significantly increased levels in *NPC1* and *SCARB2* KO cells and a trend toward an increase in *SPNS1* KO cells (Figure 4k). Finally, we set out to address whether adding exogenous GCase could augment lysosomal GCase activity within the altered lysosomal milieu of these cells, as measured using LysoFQ-GBA (Figure 4l, Figure S15). We found that delivery of Brainshuttle GCase, a fusion protein linked to an antitransferrin antibody,^54^ could rescue the deficiency in GCase activity within *SCARB2* and *SPNS1* KO cells but had a more limited effect in *NPC1* KO cells. Notably, because the BrainShuttle GCase can reach lysosomes by endocytosis after binding to the transferrin receptor it bypasses the need to bind to LIMP2.^54^ The results suggest that PD associated with variants in some lysosomal genes, including those involved in trafficking from the secretory pathway, could possibly benefit in some cases from GCase therapy. In contrast, in the case of NPC, the resulting cholesterol accumulation may be more deleterious to lysosomal GCase activity.

Looking at these collective data, disruption of *SCARB2* resembles the effects seen from disruption of *GBA1*, whereas disruption of *NPC1* and *SPNS1* gives rise to disparate changes in the end points examined. Considering these observations in the context of the time-dependent changes we observed, these results reinforce the hypothesis that, while *SCARB2* disruption directly impedes GCase lysosomal activity by preventing its transport to lysosomes, impaired function of NPC1 or SPNS1 leads to gradual changes in cellular lipid composition that impact the cellular environment in different ways. Nevertheless, impairment in either subsequently leads to a decrease in lysosomal GCase activity.

## Conclusions

Genome-wide screening using CRISPR-Cas9 in combination with large libraries of pooled sgRNA are accelerating the identification of genes with specific cellular functions.^55,56^ Here we leveraged genome-wide CRISPR-Cas9 screening, in conjunction with live cell imaging of enzyme activity, to identify a broad set of candidate modifiers of lysosomal GCase activity. To our knowledge, such genome-wide enzyme-based screening with organelle resolution using synthetic substrates is rarely pursued, perhaps due to associated technical challenges including the need for well-characterized substrates that are both highly selective and stable over time. However, the excellent dark-to-light switching of LysoFQ-GBA substrate, coupled with its linear kinetic response within various cell lines, including the H4 cells used here, allowed robust and reproducible genome-wide screening using pooled libraries of sgRNAs. Using this screen we identified 40 genes affecting lysosomal GCase activity at a FDR of <5%. While the number of genes observed precludes detailed discussion, perusal reveals many genes that have logical links to lysosomal function and GCase activity. In particular, as noted above, we found that many genes implicated in lysosomal maturation, lysosomal pH homeostasis, and lysosomal lipid composition influence GCase activity. Interestingly, we also observed *GNPTAB*, which is essential for M6P-dependent trafficking, which might suggest a direct requirement for M6P for the trafficking of GCase. However, we note that even in the *SCARB2* KO cells, though at lower levels, we still observe folded lysosomal GCase, which supports the proposal that GCase can indeed reach the lysosome—at least in part—through an M6P-independent process.^7^ In addition, the large number of genes linked to neurodegenerative diseases, especially PD, is striking and suggests that these may act upstream of *GBA1* to influence lysosomal GCase activity. Our experiments with substrates for other lysosomal enzymes indicate that such perturbations are generally not deleterious for the function of lysosomal enzymes other than GCase. Indeed, changes arising from KO of *NPC1* or *SPNS1* do not affect Cathepsin B and, surprisingly, increase the activity of GalA. These observations underscore the sensitivity of lysosomal GCase and suggest why mutations in *GBA1* might be commonly linked to PD yet exhibit relatively low penetrance. We speculate that GCase is sensitive to genetic perturbations and environmental stimuli that influence the lysosomal environment and that such sensitivity can be compounded by even relatively mild heterozygous variants in *GBA1*.

Despite the exceptional performance and reproducibility of our screening assay, there are limitations in its application. First, because LysoFQ-GBA depends on endocytosis to reach lysosomes, it is possible that some of the candidate genes we have identified here appear by virtue of their effect on the endocytosis rate rather than affecting lysosomal GCase activity. This point can be readily addressed through simple counter screening as performed here using alternative lysosomal reporters, such as Gal-BABS, which are also taken up by endocytosis. We also speculate that alternative CRISPR screening strategies that are less disruptive may enable identification of genes having essential roles in cellular function that cannot be detected using CRISPR-Cas9 due to their lethal loss of function. In particular, CRISPR-activation using patient cells, including neurons, may identify genes for which increased expression facilitates maturation or retention of GCase within lysosomes and may explain why some genes, such as *TMEM175*, which are known to influence GCase activity within neurons were not identified.^22^ The future pursuit of such studies using alternative cell types is therefore well justified in light of the results observed here and could extend or yield new findings with respect to the regulation of lysosomal GCase activity. Nevertheless, the large set of candidate modifiers of GCase activity identified here will enable the wider community to perform more targeted experiments on candidate genes of interest.

While many of the genes identified were expected based on their known functions, many others within our data set encode proteins with activities that are not well-defined. We focused on two genes that encode the only known lysosomal lipid transporters: *NPC1* and *SPNS1*. Examination of PD GWAS data revealed that certain *SCARB2* and *SPNS1* alleles, which result in decreased cellular expression, are associated with PD yet not with AD. We further showed that disruption of *SPNS1* impairs GCase activity in a time-dependent manner, consistent with loss of this transporter exerting comparatively slow changes in lysosomal lipid composition. This change leads, ultimately, to decreased levels of lysosomal GCase protein that drive the observed impairment in lysosomal GCase activity. These functional data again point to the sensitivity of GCase to lysosomal conditions. Moreover, though we observed only a slight increase in GlcSph levels in *SPNS1* KO cells, it is notable that, in recently described *SPNS1* KO mice, there are clear increases in sphingosine levels, including both GCase substrates GlcSph and GlcCer, and emergence of a LSD phenotype—pointing to the development of clear lysosomal dysfunction.^57^ While it is tempting to speculate that accumulation of the SPNS1 substrates,^42,43^ LPC and LPE, within lysosomes may itself impair GCase function, it is notable that we see a decrease in the levels of folded lysosomal GCase in *SPNS1* KO cells, which perhaps suggests that loss of SPNS1 induces a defect in the trafficking of GCase to lysosomes. Resolution of this question will require more detailed mechanistic cellular studies.^7^

In summary, our data identified *SPNS1* as a candidate genetic risk and disease progression factor for PD that influences lysosomal GCase activity. Further, while dysfunction in the endolysosomal system is well-recognized as being linked to PD,^58^ the high convergence between hits within our cell screening data and known genetic risk factors for PD indicate that GCase is particularly sensitive to perturbations in the lysosomal environment. This suggests that upstream perturbations—both genetic and environmental—may converge on GCase to impair its activity. Interestingly, the penetrance of PD among carriers of *GBA1* mutations is modest, suggesting that these mutations alone may not be sufficient to cause PD. This suggests that additional genetic impairments may be implicated in triggering the development of *GBA1*-associated PD. In that regard, we expect that the candidate modifiers of GCase described here will enhance our understanding of how these genes are linked to the lysosomal activity of GCase and, perhaps, to PD. Genes identified that have no obvious link to lysosomal function or GCase maturation may also provide clues into the roles of the corresponding protein products on these processes. Finally, because mutations in *GBA1* are the most common genetic risk factor for PD and augmentation of its activity ameliorates symptoms in PD preclinical models, we envision that an improved understanding of the regulation of lysosomal GCase activity should open new therapeutic avenues to augment its activity. Such insights may ultimately help to deliver a potentially new and much needed disease-modifying therapy for PD.

## Supplementary Material







## Notes

Supplementary figures (Figures S1–S14), detailed experimental procedures, and additional references (PDF)
Transparent Peer Review report available (PDF)
Tables of results (Tables S1–S4) (ZIP)
