# Supplementary material for: Enzyme Activity-Based Genome-wide Screening for Modifiers of Lysosomal Glucocerebrosidase Uncovers Candidate Risk Factors for Parkinson’s Disease
Source: ACS Cent Sci. 2025 Sep 3;11(10):1933–45. doi: 10.1021/acscentsci.5c00240 (PMC12550621; doi:10.1021/acscentsci.5c00240)
Supplement: Supplementary file 1 [file oc5c00240_si_001.pdf]

Supporting Information

Enzyme activity-based genome-wide screening for modifiers of lysosomal glucocerebrosidase uncovers candidate risk factors for Parkinson disease

Vinod Udayar[1,2,†], Pierre-André Gilormini[3,4,†], Julien Bryois[1,†], Alexandra Gehrlein[1], Xi Chen[4], Stephanie Sonea[3], Sha Zhu[4], Matthew C. Deen[4] , Nadia Anastasi[1], Alan E. Murphy[5,6], Nathan Skene[5,6], Manuela M. X. Tan[7], Jon-Anders Tunold[7], Filip Roudnicky[2], Wilma D.J. van de Berg[8,9], Lasse Pihlstrøm[7], David J. Vocadlo[3,4,*], Ravi Jagasia[1,*]

[1] *Roche Pharma Research and Early Development, Neuroscience and Rare Disease discovery and translational area, Roche Innovation Center Basel, 4070 Basel, Switzerland.*
[2] *Roche Pharma Research and Early Development, Therapeutic Modalities, Roche Innovation Center Basel, 4070 Basel, Switzerland.*
[3] *Department of Molecular Biology and Biochemistry, Simon Fraser University, Burnaby, BC V5A 1S6 Canada*
[4] *Department of Chemistry, Simon Fraser University, Burnaby, BC V5A 1S6 Canada*
[5] *UK Dementia Research Institute at Imperial College London, United Kingdom*
[6] *Department of Brain Sciences, Imperial College London, United Kingdom*
[7] *Department of Neurology, Oslo University Hospital, Oslo, Norway*
[8] *Department of Anatomy and Neurosciences, Section Clinical Neuroanatomy and Biobanking, Vrije Universiteit Amsterdam, Amsterdam, 1081 HV, Netherlands.*
[9] *Amsterdam Neuroscience, program Neurodegeneration, Amsterdam, Netherlands.*

[†] *Equal contribution*
[*] Equal Co-corresponding Authors: E-mail: dvocadlo@sfu.ca, ravi_jagasia@roche.ca,

Supplementary Methods

Cell culture

H4 cells (ATCC HTB-148) were maintained in a mixture DMEM/F12 (1:1) (ThermoFisher, #11320082) supplemented with p/s and 10 % FBS (Gibco). Cells were cultured in T75 or T175 flasks and incubated at 37 °C, 5 % CO_2. When reaching confluence, cells were incubated with a mixture of Trypsin/EDTA until fully detached. Cells were then diluted with culture media, centrifuged (150 x g, 10 min) and resuspended in fresh media and re-seeded at the desired concentration for maintenance or the desired experiments.

GCase activity-based assay in live cells (microscopy)

The measurement of GCase lysosomal activity in live H4 cells using LysoFQ-GBA was performed in a way similar to what we have described before.[1] The cells are seeded in the appropriate vessel (96- or 384-well plate, Corning 4680 or 4681 respectively). Cell number for seeding is adjusted as a function of the desired experiment. After seeding, the plate is centrifuged, (150 x g, 30 seconds) and incubated overnight at 37 °C, 5 % CO_2. The GCase live-cell assay is performed after the cells have been submitted to any treatment or perturbation desired as described in the multiple experiments below. When appropriate, cells are treated with 10 µL (96-well plate) or 5 µL (384-well plate) of a solution of LysoFQ-GBA in culture media. The concentration for dispensing is 10 times the final desired concentration in the well (50 µM dispensing solution for a final concentration of 5 µM for example). After dispensing, the cells are centrifuged (150 x g, 30 sec) and incubated for 1 h at 37 °C, 5 % CO_2. Following the incubation, the cells are washed 3 times with 100 µL (96-well plate) or 50 µL (384-well plate) of sterile PBS and then dispensed with 100 µL (96-well plate) or 50 µL (384-well plate) of Phenol-red free MEM supplemented with 10 % FBS, and 10 µM of selective GCase inhibitor AT3375 and Hoestch reagent (nuclei staining). The presence of AT3375 in the reading medium enables to stop any further turnover of the quenched substrate already in the cells and to prevent signal deviation over the time of reading the plate. Upon dispensing of the reading medium, cells are centrifuged (150 x g, 30 sec) and incubated for 20 minutes at 37 °C, 5 % CO_2. The plate is then imaged.

The high-content microscopes used in this manuscript are:

- ImageXpress Micro XLS high-content imager (Molecular Devices) connected to environmental control (37 °C, 5 % CO_2). Image acquisition was carried out using a 40X objective. For each well, four to 9 sites were imaged using the DAPI and FITC channels (unless further specifications). Before acquisition, the focus was adjusted for both channels and the exposure times were set to 50 ms for DAPI and 300 - 500 ms for FITC.
- Opera Phenix Plus (Perkin Elmer/Revvity) connected to environmental control (37 °C, 5 % CO_2). Image acquisition was carried out using a 63X objective. For each well, 24 to 30 sites were imaged. Image analysis were performed with PerkinElmer/Revvity's Harmony 5.1 software.

GCase activity-based assay in live cells (cytometry)

Cells were seeded were seeded in the appropriate vessel (6-, 12-, 24-, or T175 flasks). Cell number for seeding is adjusted as a function of the desired experiment. After seeding, the plate is centrifuged, (150 x g, 30 seconds) and incubated overnight at 37 °C, 5 % CO_2. The GCase live-cell assay is performed after the cells have been submitted to the desired treatment or perturbation desired as described in the multiple

experiments below. When appropriate, growing medium is replaced by a solution of LysoFQ-GBA in fresh medium (2.5 µM unless other specification). Cells are then incubated for 1 h (unless specified otherwise) at 37 °C, 5 % CO_2. After incubation, medium was removed and cells were washed twice with cold sterile PBS. Then, cells were trypsinized and incubated until all the cells were detached. Trypsin was diluted with PBS and transferred into a 5 mL cytometry tube (Corning, #352028). Cells were then centrifuged (150 x g, 10 minutes). Supernatant was discarded and the cells were resuspended in Phenol-red free MEM supplemented with 10 % FBS, and 10 µM of selective GCase inhibitor AT3375. When required, the phenol red-free medium was supplemented with Hoetsch or Calcein Orange. Cells were then kept on ice until cytometry analysis or sorting. Analytic cytometry was performed with a BD LSRFortess X-20 Cell Analyzer 4-laser | 16-Colour [2B/3R/5YG/6V], sorting was performed with a BD FACSAria Fusion Cell Sorter 4-Laser | 16-Colour [2B/3R/5YG/6V] or a benchtop SH800S cell sorter. Cytometry data was analyzed using FlowJo™ v10.8 (BD Life Sciences).

Targeted pilot screen

The procedure for the screen was adapted from Piccioni.[2] Briefly, H4 cas9 cells were cultured as described above in T175 Flasks. For transduction 7.5 X10^6 cells were plated in T175 flasks a day prior to transduction. On the day of transduction (Day 1) medium was supplemented with freshly prepared transduction medium containing lentiviral library (Functional Titer: 1.18E+08 TU/ml; 60 ul per T175 flask, target MOI: 0.5, final medium volume: 20 ml) with polybrene (6 µg/mL final) in culture medium. Cells were incubated overnight. The next day (Day 2) 10 ml fresh medium was added to the flask. On Day 4, cells were split into multiple flasks. On Day 8, culture medium was replaced by fresh medium supplemented with Puromycin (3.5 µg/mL). Sample cells were seeded in a 96 well-plate to determine transduction efficiency. Actual achieved MOI was calculated to be approx. 0.5.

After 3 days incubation with puromycin, cells were trypsinized and pooled together. Then they were seeded back in batches of different seeding density – highest density aiming to be sorted on the earliest, lowest density aiming to be sorted the latest. The next day, the highest density cells were treated with LysoFQ-GBA as described in the corresponding section. In parallel, non transduced cells were also incubated with LysoFQ-GBA and subsequently stained with Hoechst reagent in order to be used as an internal control for sorting. The low activity cells (low FITC signal) were collected and seeded back. After expansion for 10 days, half the cells were centrifuged and the cells pellet were frozen prior to sequencing. The other half was submitted to GCase activity assay with LysoFQ-GBA and sorted again for a second enrichment. The process was repeated a third time and all the collected cell pellets were subsequently submitted to Next-Generation sequencing.

Sequences for the custom sgRNA library are available in the Supplementary Table S1

Genome-wide screen

The genome-wide screen was performed following a similar protocol as the targeted pilot screen, on a larger scale. 100 million H4-Cas9 cells were transduced with the genome-wide library. The cells went through three rounds of enrichment based on GCase lysosomal activity and cell pellets were collected and frozen after each one before being sequenced.

Sequences for the genome wide human sgRNA library are available in the Supplementary Table S4.

Sequencing and Data analysis - Targeted Pilot Screen

DNA Isolation:

DNA was isolated following the manufacturers instructions using the Qiagen DNeasy blood & tissue kit and diluting the input to 1 Mio cell / column. Concentrations were measured with Lumiprobe Pico488.

Enrichment PCR + First-step PCR:

The isolated DNA was normalized to 125 ng/µl and 1.25 µg (10 µl) of template DNA was PCR amplified in several replicates (depending on the total amount of template DNA), using the KAPA HiFi HotStart Polymerase (Cat. KR0369, Roche). The target-specific enrichment PCR primers (Forward primer GGCCTATTTCCCATGATTCC*T, Reverse primer TCTTTCCCCTGCACTGTAC*C, * = PTO bond) were applied with a final concentration of 5 µM. Enrichment PCR occurred in a thermal cycler using the following program: 3 minutes at 95°C; 35 cycles of 20 sec. at 98°C, 15 sec. at 64°C, 20 sec. at 72°C, and 2 min. at 72°C. The resulting PCR products from the enrichment PCR were then pooled, 1:100 diluted and subsequently amplified in triplicates (first-step PCR) using the same polymerase as in the enrichment PCR. First-step PCR primers with Nextera Illumina adaptors were applied with a final concentration of 0.3 µM. First-step PCR was performed using the following thermal cycler program: 3 minutes at 95°C; 20 cycles of 20 sec. at 98°C, 15 sec. at 64°C, 20 sec. at 72°C, and 2 min. at 72°C. After cycling, the 3 PCR reactions per sample were pooled.

Libraries:

Samples (1st Step PCR Products) were purified using NGS Clean Beads, quantified using pico488 and diluted to 2.5ng/ul. Input for 2nd Step PCR was 12.5 ng. Optimal PCR conditions for 2x HOT FIREPol MultiPlex Mix (Solis Biodyne) and Nextera XT v2 Index Primers were applied (10 PCR Cycles). Samples were purified using NGS Clean Beads, quantified using pico488 and pooled equimolarly. The pool was purified using NGS Clean Beads, quantified using pico488 and library size was checked on Bioanalyzer (Agilent).

Sequencing:

Subsequently the PCR libraries were sequenced on two Illumina NextSeq 500/550 platforms using two high output sequencing chips in single-end mode (1x75bp) and combinatorial dual indexes.

Bioinformatics:

The produced single-end reads which passed Illumina's chastity filter were subject to de-multiplexing with zero mismatches using Illumina's bcl2fastq software version v2.20.0.422. The quality of the reads was checked with the software FastQC version 0.11.8 and sequencing reads that fell below an average Q-score of 20 or had any uncalled bases (N) were removed from further analysis. The locus specific primers were trimmed from the sequencing reads with the software cutadapt v3.2, keeping only trimmed reads of length 19 or 20 bases. The mapping software BBMap v38.86 was used to map the surviving reads in semiperfect mode to the provided sgRNA reference set and calculate the read-depths for each sgRNA sequence. Differential expression analysis was performed with the R package edgeR v3.28.1 (discarding any sgRNAs with less than 10 mapped reads). Normalization factors were derived to estimate dispersion and fit the data with a generalized linear model to be able to calculate differential expression and derive fold changes and their corresponding significance. Geneset analysis was performed with the R package

limma v3.42.2 converting the known gene symbols to entrez ids and filtering with a false discovery rate of 0.05.

DNA extraction, library construction, sequencing and data analysis described in these sections were performed by Microsynth AG (Balgach, Switzerland).

Sequencing and Data analysis - Genome-wide screen

DNA Isolation:

DNA was isolated following the manufacturers instructions using the Geneaid Blood/Cell DNA Maxi kit and diluting the input to 1 Mio cell / column. Concentrations were measured with Lumiprobe Pico488.

Enrichment PCR + First-step PCR:

The isolated DNA was normalized to 150 ng/µl and 1.5 µg (10 µl) of template DNA was PCR amplified in 80 replicates (total amount of template DNA: 120 µg), using the KAPA HiFi HotStart Polymerase (Cat. KR0369, Roche). The target-specific enrichment PCR primers (Forward primer GGCCTATTTCCCATGATTCC*T, Reverse primer TCTTTCCCCTGCACTGTAC*C, * = PTO bond) were applied with a final concentration of 5 µM. Enrichment PCR occurred in a thermal cycler using the following program: 3 minutes at 95°C; 35 cycles of 20 sec. at 98°C, 15 sec. at 64°C, 20 sec. at 72°C, and 2 min. at 72°C. The resulting PCR products from the enrichment PCR were then pooled, 1:100 diluted and subsequently amplified in triplicates (first-step PCR) using the same polymerase as in the enrichment PCR. First-step PCR primers with Nextera Illumina adaptors were applied with a final concentration of 0.3 µM. First-step PCR was performed using the following thermal cycler program: 3 minutes at 95°C; 20 cycles of 20 sec. at 98°C, 15 sec. at 64°C, 20 sec. at 72°C, and 2 min. at 72°C. After cycling, the 3 PCR reactions per sample were pooled.

Libraries:

Samples (1st Step PCR Products) were purified using NGS Clean Beads, quantified using pico488 and diluted to 2.5ng/ul. Input for 2nd Step PCR was 12.5 ng. Optimal PCR conditions for 2x HOT FIREPol MultiPlex Mix (Solis Biodyne) and IDT for Illumina DNA/RNA UD Indexes Primers were applied (10 PCR Cycles). Samples were purified using NGS Clean Beads, quantified using pico488 and pooled in equimolar amounts. The pool was purified using NGS Clean Beads, quantified using pico488 and library size was checked on Fragment analyzer (Agilent).

Sequencing:

Subsequently the PCR libraries were sequenced on an Illumina NovaSeq 6000 platform using two lanes on a S2 sequencing chip in paired-end mode (2x51bp) and unique dual indexes.

Bioinformatics

The produced paired-end reads which passed Illumina's chastity filter were subject to de-multiplexing with zero mismatches using Illumina's bcl2fastq software version v2.20.0.422. The quality of the reads was checked with the software FastQC version 0.11.8 and sequencing reads that fell below an average Q-score of 20 or had any uncalled bases (N) were removed from further analysis. Only the first read of each

paired-read was kept and the second read was discarded as it contained redundant information. The locus specific primers were trimmed from the sequencing reads with the software cutadapt v3.2, keeping only trimmed reads of length 19 or 20 bases. The mapping software BBMap v38.86 was used to map the surviving reads in semiperfect mode to the provided sgRNA reference set and calculate the read-depths for each sgRNA sequence. Differential expression analysis was performed with the R package edgeR v3.28.1 discarding any sgRNAs with less than 10 mapped reads. Normalization factors were derived to estimate dispersion and fit the data with a generalized linear model to be able to calculate differential expression and derive fold changes and their corresponding significance. Geneset analysis was performed with the R package limma v3.42.2 converting the known gene symbols to entrez ids and filtering with a false discovery rate of 0.05.

DNA extraction, library construction, sequencing and data analysis described in these sections were performed by Microsynth AG (Balgach, Switzerland).

Genetic enrichment of GCase regulators

We used MAGMA[3] to test whether GCase regulators from our crispR screen (FDR<5%) were enriched in disease risk. We used the following window size to aggregate GWAS SNP pvalues around each gene into gene-level statistics: 35kb upstream to 10kb downstream of the gene. We then tested whether the GCase regulators were enriched in genetic risk among all genes tested in our CrispR screen ("--settings gene-include" in MAGMA). We obtained GWAS summary statistics for Parkinson's disease risk from 23andMe and used publicly available GWAS summary statistics for schizophrenia.[4]

Genetic co-localization analysis

We used the coloc R[5] package to test whether the GWAS signal at the SPNS1 locus was consistent with eQTL signals affecting SPNS1 in microglia from post-mortem human brains.[6,7] We included all SNPs in linkage disequilibrium (LD; $R^2 > 0.1$ based on the 1000 Genomes Project European cohort) with the lead SNP (rs2904880) at the SPNS1 locus.[8] The analysis was performed using effect size estimates (beta values) rather than p-values to enhance the precision of the co-localization inference.

Generation of H4 Cas9 KO lines

H4 GBA1 KO cells were generated as previously described by Jong and Gehrlein *et al.* 2024.[9]

CRISPR/Cas9 gene editing was performed to generate SCARB2, NPC1 and SPNS1 KO H4 cell lines: In brief, H4 GBA KO cells were seeded at 1E5 cells/well into a 6 well-plate and transfected with RNPs at a 1.8:1 ratio (sgRNA:Cas9 nuclease) by lipofection. Lipofection reagents were purchased from Thermo Fisher Scientific (Lipofectamine™ CRISPRMAX™ Cas9 Transfection Reagent #CMAX00015). Media was changed to full growth media after 18h and cells were subjected to limiting dilution to obtain monoclonal cell populations.

Synthetic sgRNAs were purchased from Synthego:

SCARB2:

CTACAGCCTTCTGGAAGACC

NPC1:

CAACUGGAUUGGUUGUGACC

GAGAAGAAAAUGACACAGCC

UGCGCUGACAGGGUCACAGC

SPNS1:

AGUGUUCAUCUCCAGUUACA

AUCGGAAGUAUCUCAUGUGC

AGCUUGGGGCCUCACCUCUC

Time-dependent KO

GCase activity in cell lysates

To assess GCase bulk activity from whole cell lysate, H4 cells from all lines were seeded at 5E4 cells/well into a 96-well plate and maintained at 37 °C, 5% CO_2, 85% humidity until they reached confluency. Cells were washed once with PBS and lysed in 30 µl lysis buffer (0.05 M citric acid, 0.05 M KH_2PO_4, 0.05 M K_2HPO_4, 0.11 M KCl, 0.01 M NaCl, 0.001 M $MgCl_2$, pH 6.0 with 0.1% (v/v) TritonX-100, supplemented with freshly added protease inhibitor). 10 µl of cell lysate were mixed with 10 µl of 10 mM resorufin-β-glucopyranoside and baseline fluorescence was measured at t_0 immediately. The build-up of fluorescent product (resorufin) was measured after incubation for 2 h at 37 °C (λ_{ex} = 535 nm and λ_{em} = 595 nm) indicating GCase activity. Data was acquired at a multimode plate reader (SpectraMax Paradigm, Molecular Devices; Software: SoftMax Pro 7). Data was normalised to WT cells.

GCase protein levels in cell lysates

To assess GCase bulk protein levels from whole cell lysate, a customized AlphaLISA assay was performed. H4 cells from all lines were seeded at 5E4 cells/well into a 96-well plate and maintained at 37 °C, 5% CO_2, 85% humidity until they reached confluency. Cells were washed once with PBS and lysed in 30 µl GCase lysis buffer (0.05 M citric acid, 0.05 M KH2PO4, 0.05 M K2HPO4, 0.11 M KCl, 0.01 M NaCl, 0.001 M MgCl2, pH 6.0 with 0.1% (v/v) TritonX-100, supplemented with freshly added protease inhibitor). A 2-fold serial dilution of imiglucerase (Genzyme) ranging from 1250 pM - 2.4 pM was generated as a standard curve. 10 µl of samples or standards were incubated for 4 hours at RT in the dark with 1 nM of biotinylated Roche-hGCase-1/23 and 20µg/ml of Roche-hGCase-1/17-conjugated Acceptor beads (beads:antibody 50:1). Subsequently, 40 µg/ml of AlphaScreen Streptavidin Donor beads (Perkin Elmer, #6760002) were added and incubated 1 hour at RT in the dark. Plate was read on a Tecan Spark plate reader with ex: 680 nm and em: 520-620 nm. GCase concentration was calculated based on a sigmoidal non-linear regression of the imiglucerase standard and then normalized to total protein concentration.

Immunoblots analysis

To check protein levels in the various H4 KO lines, Western Blot was performed. In brief, 1E6 cells were lysed in lysis buffer (0.05 M citric acid, 0.05 M KH_2PO_4, 0.05 M K_2HPO_4, 0.11 M KCl, 0.01 M NaCl, 0.001 M $MgCl_2$, pH 6.0 with 0.1% (v/v) TritonX-100, supplemented with freshly added protease inhibitors

(cOmplete; Roche, #11873580001), and 20 µg of total protein were subjected to SDS-PAGE (Invitrogen NuPAGE system). Semi-dry transfer at 23 V for 6 min was used to transfer proteins onto a nitrocellulose membrane, which was blocked in Fast Blocking Buffer (ThermoFisher # 37575) and subsequently incubated with primary antibody at 1/1000 o/n at 4 °C (primary antibodies: rb pAb anti-SPNS1, Thermo #PA5-59813; rb mAb anti-NPC1 Abcam #ab134113; ms mAb anti-GCase, Abcam #ab55080; rb mAb anti-LIMP2, Abcam #ab176317). After 3 washes in TBST, HRP-labelled secondary antibody was incubated for 1.5 hrs at RT at 1/10000 in Fast Blocking Buffer. Proteins were detected using the SuperSignal West Dura Extended Duration Substrate Kit (ThermoFisher). Images were taken using the ChemiDoc MP Imaging System (BioRad).

GCase and LAMP1 immunolabelling and cellular localization

To assess GCase cellular localization, H4 cells were seeded at 5E4 cells/well into an imaging-compatible 96-well plate (Phenoplate, Revvity # 6055302). Cells were fixed in 4% paraformaldehyde (20 min, RT). Fixed cells were washed in PBS three times and incubated in ICC antibody diluent [0.1% Saponin (filtered) and 1% BSA in PBS] containing primary antibody overnight at 4 °C (antibodies: mouse anti-human GCase clone 1/17, (Roche, freely available through the MJFF); rabbit anti-LAMP1 clone D2D11 (Cell Signalling Technology), #9091). Wells were washed three times with PBS, then covered with aluminum foil and incubated for 2 hrs with secondary antibody diluted in ICC antibody diluent at RT. Subsequently, wells were washed three times with PBS and counterstained with DAPI to label the nuclei. Plates were imaged using a 63X water objective at Opera Phenix Plus (Perkin Elmer; Software: Harmony 5.1).

LAMP1-positive spots were identified and classified into “average sized” and “enlarged” lysosomes based on their morphology using Harmony software. Subsequently, GCase-positive spots were identified and number of spots within LAMP1-positive “average sized” spots were quantified.

GlcSph measurements

To assess bulk GlcSph levels, H4 cells from all lines were seeded at 2E4 cells/well into a 96-well plate and maintained at 37 °C, 5% CO_2, 85% humidity until they reached confluency. Cells were washed once with PBS and dry cell layer was deep frozen at -80 °C. Cells were thawed and subsequently lysed by adding distilled water and methanol containing internal analyte standards. Samples were evaporated to dryness, reconstituted in acetonitrile/water 90/10 (v/v) with 1% DMSO and analysed by LC-MS/MS. Lipids were simply quantified using the peak area ratio analyte/internal standard (= response). Data acquisition and analysis was done using MassLynx V4.1 SCN950 (Copyright 2015 Waters Inc.).

Liquid chromatography-mass spectrometry analysis of GlcSph

Analytes and internal standards were purchased from Avanti Polar Lipids: D-glucosyl-β-1-1’-D-erythro-sphingosine (GlcSph (d18:1); #860535) and D-glucosyl-β-1-1’-D-erythro-sphingosine-d5 as internal standard 1 (GlcSph-d5 (d18:1); #860636); D-galactosyl-β-1-1’-D-erythro-sphingosine (GalSph (d18:1); #860537) and D-galactosyl-β-1-1’-D-erythro-sphingosine-d5 as internal standard 2 (GalSph-d5 (d18:1); #860637).

For chromatography HPLC grade solvents as well as Millipore water was used. Acetonitrile (LiChrosolv #1.00030) and methanol (LiChrosolv #1.06007) were obtained from Supelco (Merck), ammonium acetate for mass spectrometry was purchased by Sigma-Aldrich (#73594).

Stock solutions for analytes and internal standards were prepared at 1 mM in DMSO and kept at -20 °C. For further spiking solutions acetonitrile/water 9/1 (v/v) was used as solvent. Calibration solutions were prepared by serial dilution in acetonitrile/water 9/1 (v/v) containing 2% DMSO. The concentration range was from C1 = 10 µM to C9 = 0.0039 µM. Final calibration samples were made in a pooled tissue homogenate and prepared exactly the same way as tissue samples in order to avoid suppression effects deriving from the biological matrix.

Analysis was conducted on a LC-MS-MS system consisting of a Waters Xevo-TQ-S mass spectrometer connected to a complete Waters Acquity I-class UPLC system with a flow through needle sample manager using a mixture of acetonitrile/methanol/water 40/40/20 (v/v/v) as wash solvent. The auto-sampler temperature was set to 15 °C. The Xevo TQ-S instrument operated in positive ion electrospray mode with both quadrupoles tuned to unit mass resolution using nitrogen as nebulization- and desolvation gas. The nebulizer gas flow was set to 150 l/h and the desolvation gas flow to 800 l/h with a temperature of 500 °C. Argon was used as collision gas at a flow rate of 0.15ml/min. Analytes and internal standards were detected by multiple reaction monitoring mode (MRM) following the transitions m/z 462.3 to 282.3 and m/z 467.3 > 287.3 at a cone voltage of 30 V and a collision energy of 18 V.

Samples were analysed on a BEH glycan amide column (100 x 2.1 mm, 1.7 µm particle size, purchased from Waters, Switzerland) with a flow rate of 0.25 ml/min and an oven temperature of 30 °C. Eluent A consisted of 100mM ammonium acetate and for eluent B acetonitrile was used. Glycospecific separation was achieved by isocratic elution with 90% B followed by a washing step with 10% B and column reconditioning. The overall analysis time was 12 min.

Lysosomal pH measurement

Our protocol was adapted from the procedure previously reported by Hu *et al.*[10] Briefly, cells were seeded in a 96 well-plate and incubated overnight. The next day, cells were loaded with CF-555 dextran and pHrodo^{TM} Green Dextran 10,000 MW (Thermo Fisher, #P35368) (20 µg/mL) overnight. The next day, cells were washed and fresh medium was dispensed to the cells and incubated for 1 hour. Bafilomycin A was subsequently added to the control wells and cells were incubated for an additional 2 hours. Then medium was replace by phenol-red free medium containing Hoechst for 20 minutes before being imaged as described above.

Supplementary tables

Table S1 content:

List of the sgRNAs targeting the 1496 genes (8 sgRNA per gene) and their corresponding scores in the targeted CRISPR screen.

List of the 1496 genes selected for the pilot screen and their corresponding scores in the targeted CRISPR screen (consolidated from the 8 sgRNAs).

Table S2 content:

List of the sgRNAs re-ordered for the arrayed secondary screen with the corresponding scores from the targeted screen. The four most potent sgRNAs for each gene selected in the targeted screen were re-ordered in a plate format and subsequently assessed.

Table S3 content:

Detailed results of the secondary arrayed GCase activity screening.

Table S4 content:

List of the sgRNAs targeting the whole genome for the genome-wide screen (4 sgRNAs per gene) and their corresponding scores in the genome-wide screen.

List of the genes and their corresponding scores in the targeted CRISPR screen (consolidated from the 4 sgRNAs).

Supplementary Figures

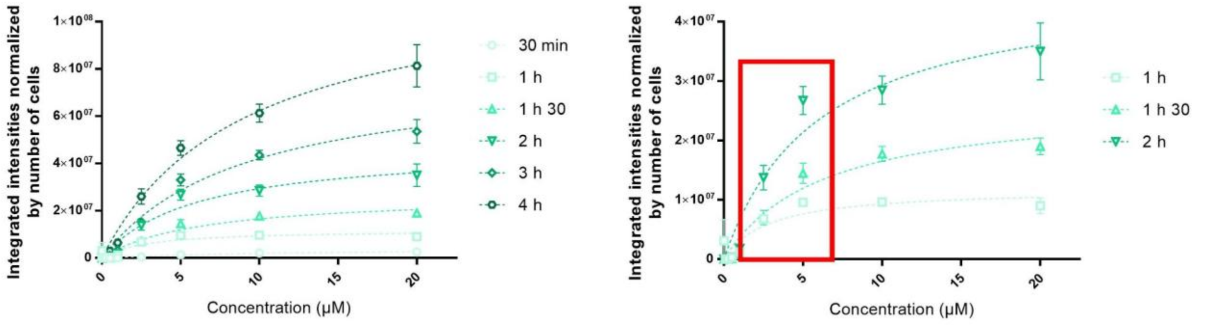

Figure S1 – LysoFQ-GBA dose- and time- response in H4 cells, readout with fluorescent microscopy. H4 cells were treated with a range of incubation times and concentrations of LysoFQ-GBA in order to determine the region of linear response allowing a quantitative measurement of enzymatic activity. Fluorescence was imaged and quantified by fluorescence microscopy.

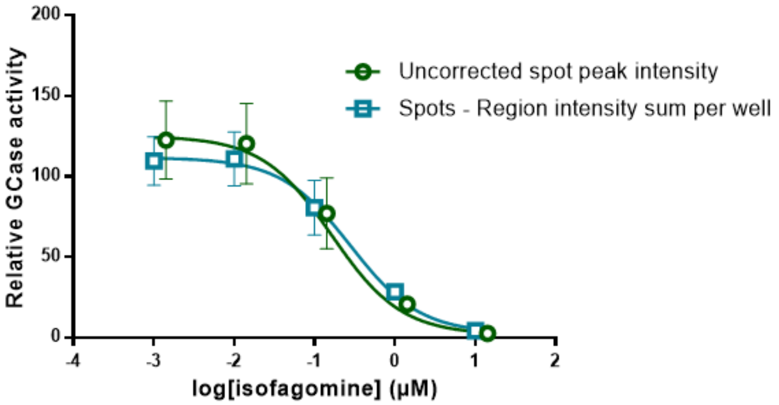

Figure S2 – IC_{50} curve of isofagomine in H4 cells. H4 cells were treated with a range of concentration of isofagomine for 1 hour. Then, cells were treated with LysoFQ-GBA, imaged, and the resulting activity was quantified and normalized for the untreated control cells. Fluorescence was imaged and quantified by fluorescence microscopy.

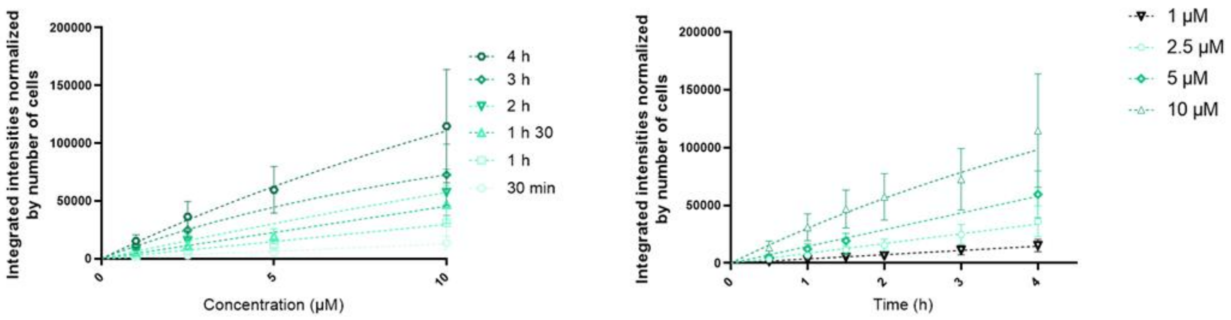

Figure S3 – LysoFQ-GBA dose- and time- response in H4 cells, readout with fluorescent cytometry. H4 cells were treated with a range of incubation times and concentrations of LysoFQ-GBA in order to determine the region of linear response allowing a quantitative measurement of enzymatic activity. Fluorescence was analyzed and quantified by fluorescent cytometry.

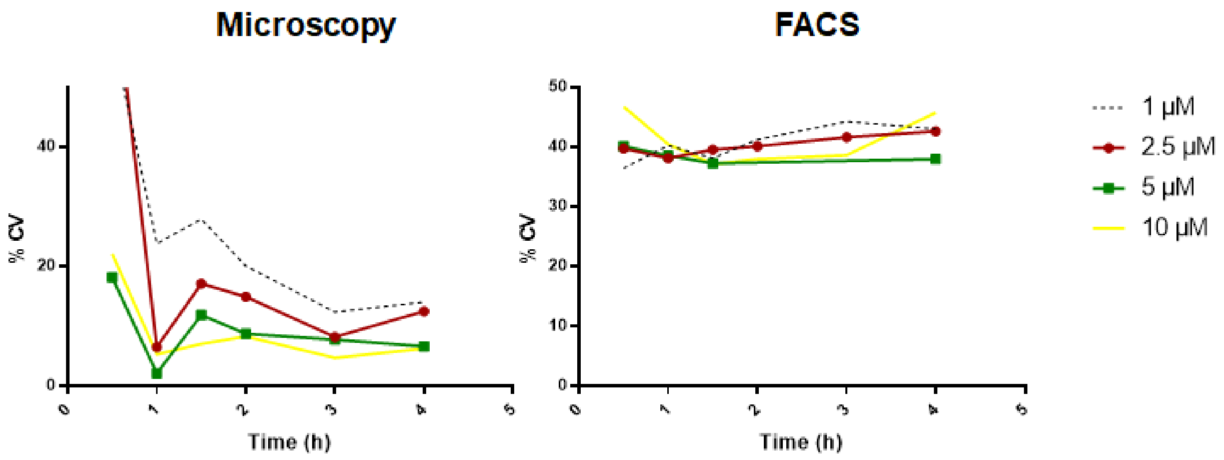

Figure S4 – Signal variability in H4 cells with fluorescence microscopy and with cytometry (FACS) readout. Evolution of %CV as a function of incubation time and concentration of LysoFQ-GBA in H4 cells and as a function of the readout technique: microscopy (left panel) or fluorescent cytometry (right panel).

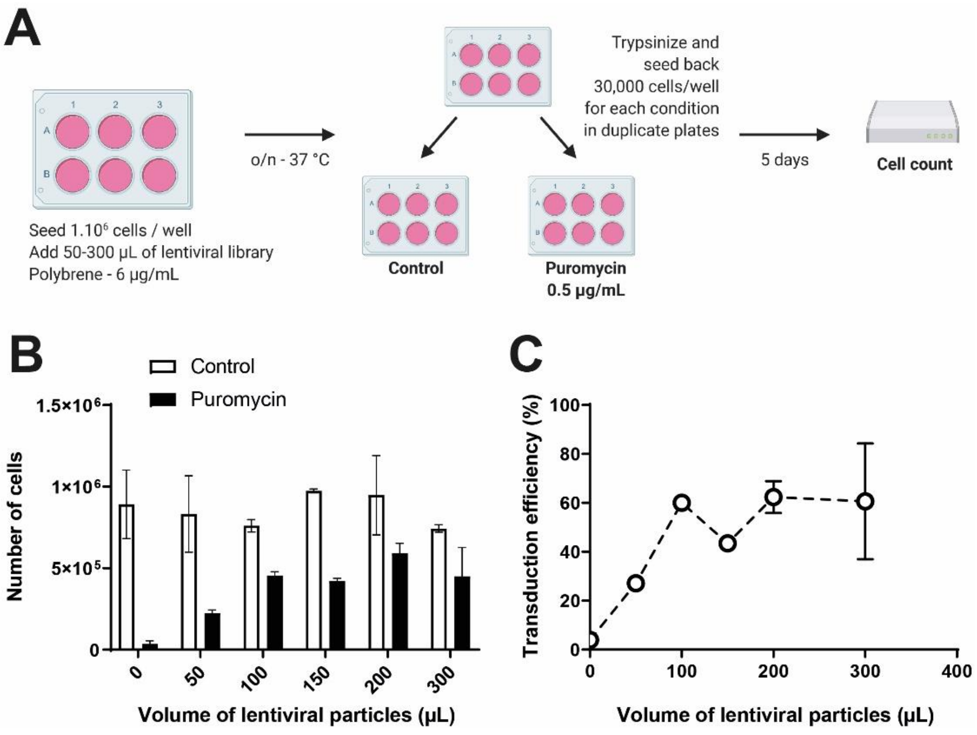

Figure S5 – Conditions optimization in H4 cells prior to the CRISPR-pooled screen: (**A**) Schematic workflow of transduction optimization. (**B**) A small sample of H4 Cas9 cells were transduced following the procedure described above with a range of lentiviral particles concentration, and then seeded back in presence or absence of Puromycin (0.5 µg/mL, optimal concentration to kill all the non-transduced cells in 3 days). Cells were counted after 5 days of incubation with Puromycin. (**C**) Transduction efficiency was determined as the ratio of the number of cells remaining after puromycin treatment over the number of cells in the corresponding untreated controls.

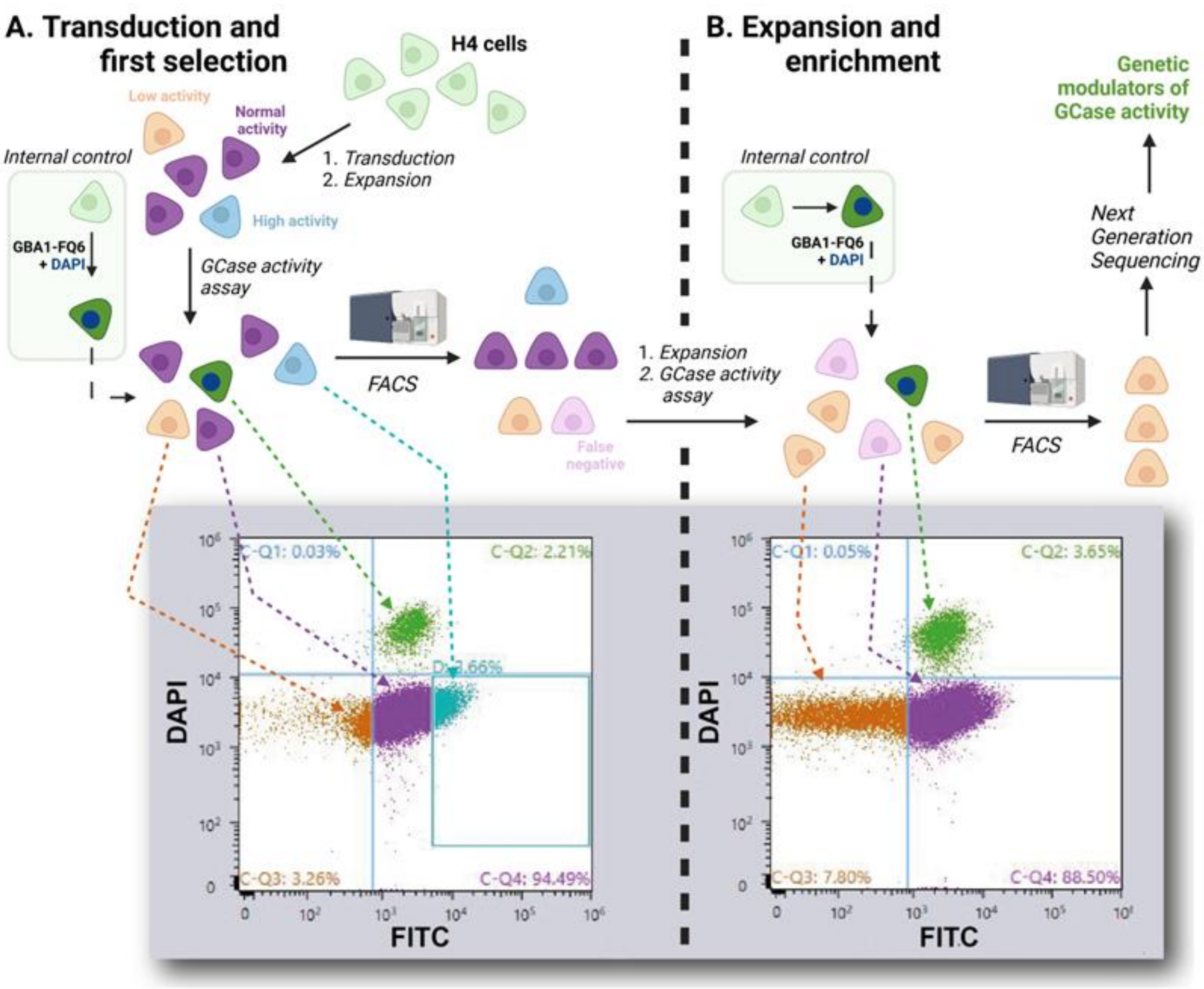

Figure S6 – Pilot screen in H4 Cas9 cells: Schematic representation of the fluorescence assisted sorting strategy used post transduction alongside representative cytometry analysis of the resulting cell populations treated with LysoFQ-GBA before (Panel A - Left) and after (Panel B - Right) enrichment in the fraction with a low GCase activity (low FITC signal).

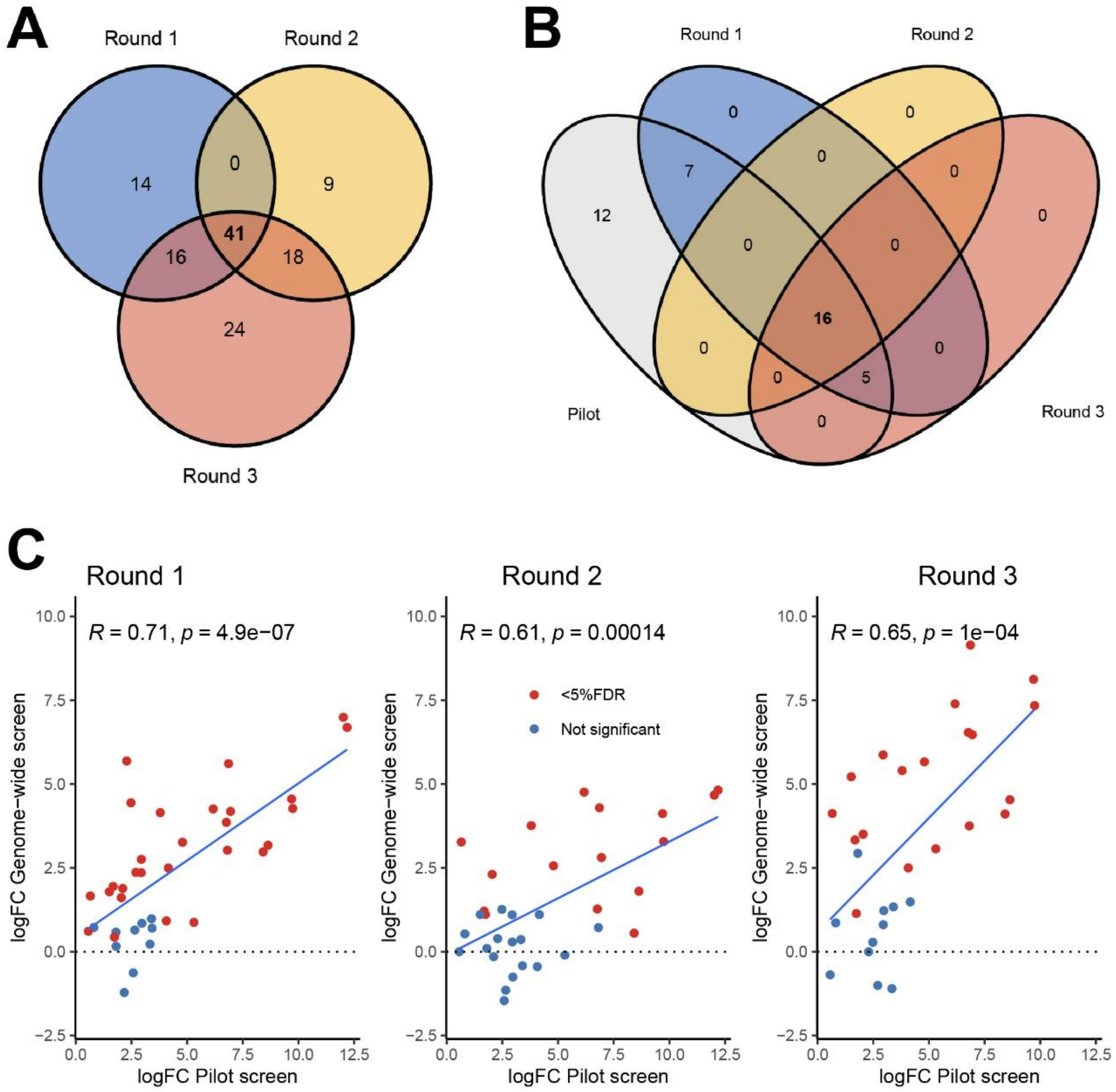

Figure S7: **Rounds of enrichment**. (A) Overlap of significant hits (5 % FDR) in different rounds of enrichment – Genome-wide screen. (B) Number of Pilot hits that replicate in genome-wide screen (5 % FDR). (C) Results are comparable between the second round of the pilot Screen and the three rounds of the genome-wide screen.

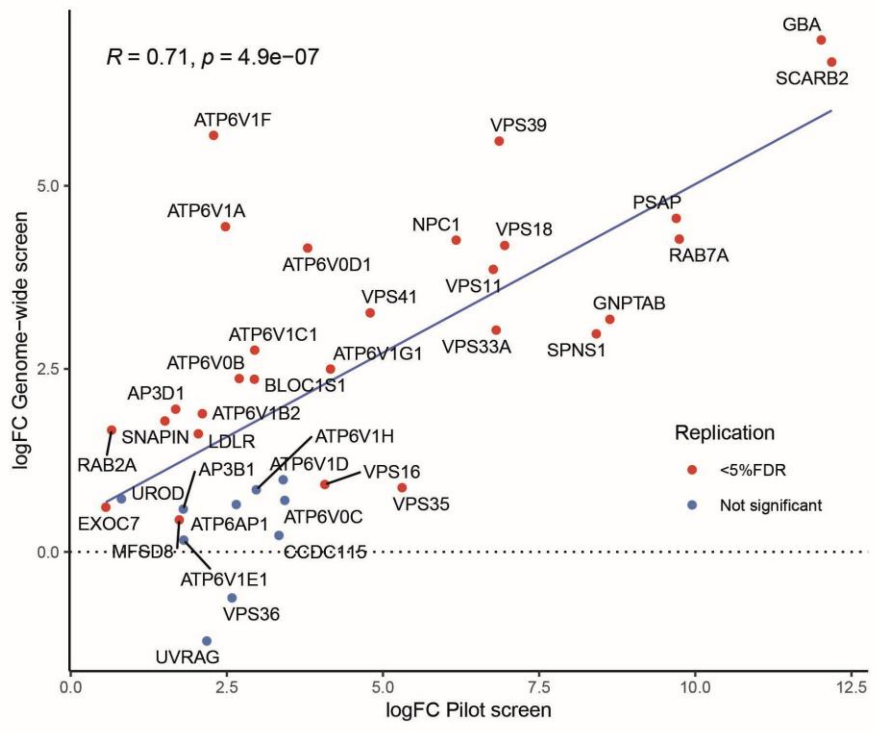

Figure S8 – Correlation between the logFC of the most significant identified genes in the pilot screen and the genome-wide screen.

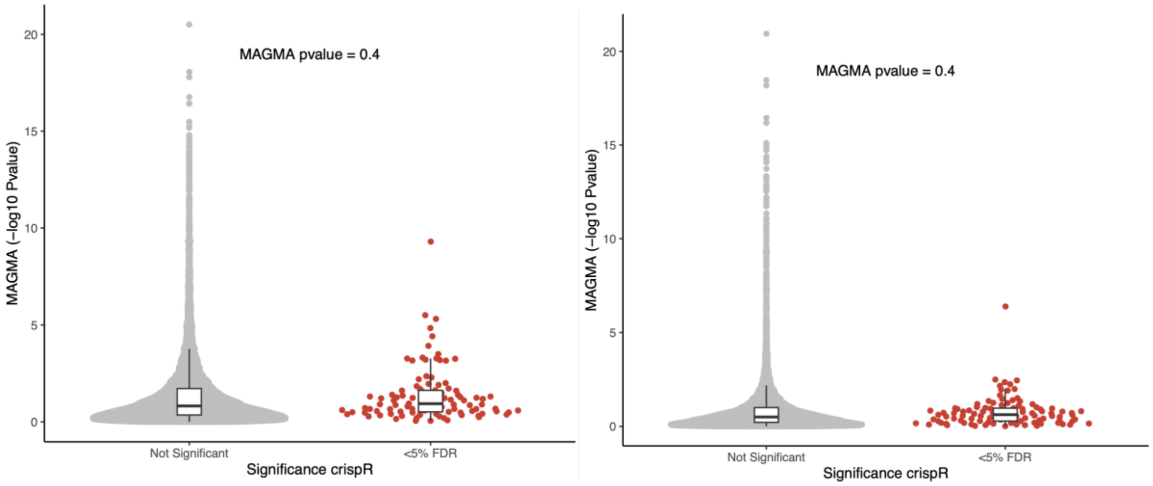

Figure S9 – Genetic enrichment of GCase regulators with Schizophrenia and Alzheimer's disease: GWAS genetic enrichment of significant GCase CRISPR screen hits (<5% FDR) using MAGMA for Schizophrenia and Alzheimer's disease.

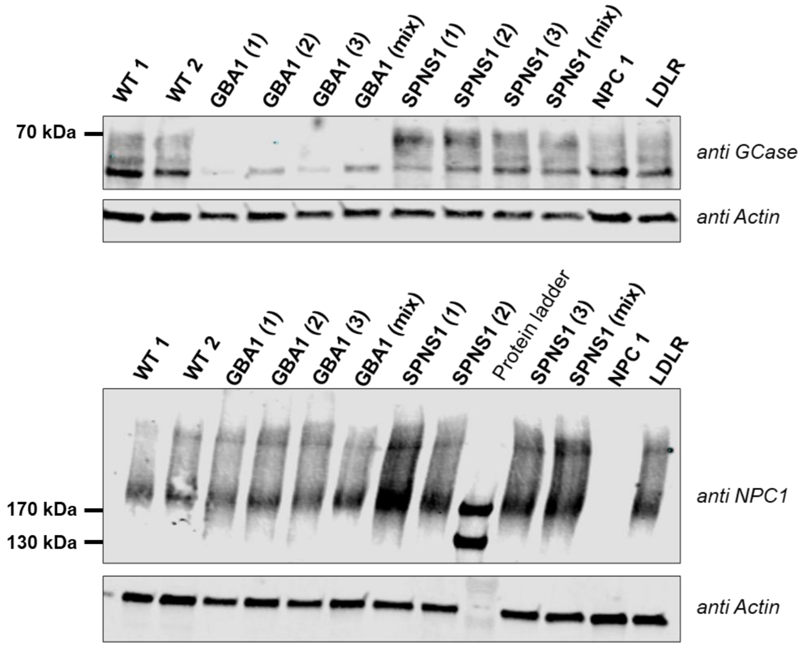

Figure S10 – Immunoblot analysis of polyclonal CRISPR KO cell lines: Each number correspond to a different sgRNA. Immunoblots have been performed with antiGcase antibody (top panel) or anti NPC1 antibody (bottom panel).

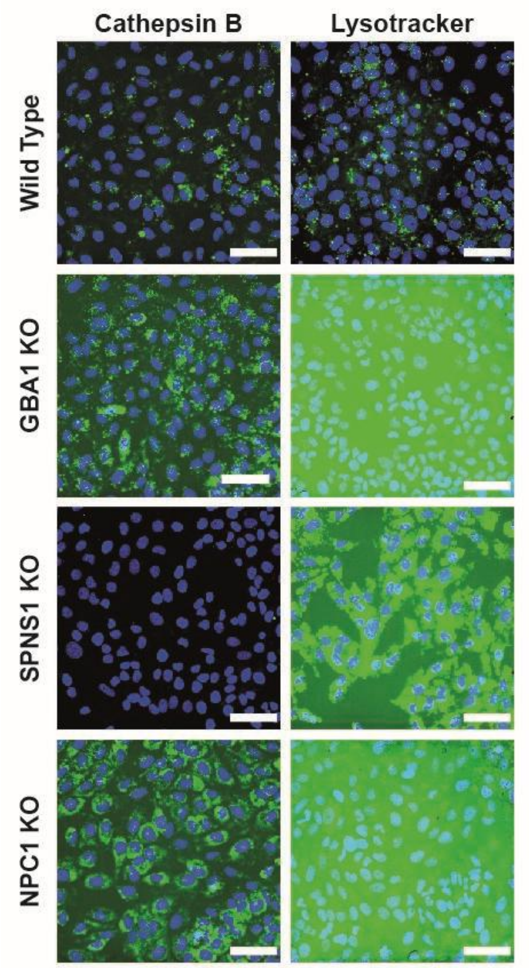

Figure S11 – Representative micrographs upon treatment of KO cell lines with Magic Red (Cathepsin B activity) or Lysotracker Green: Wild Type, GBA1 KO, SPNS1 KO and NPC1 KO H4 Cas9 cells were treated with Magic red or Lysotracker Green for an hour. The substrate was then washed away and cells were imaged with a High-content microscope (ImageXpress, Molecular Devices). Scale bar: 50 µm.

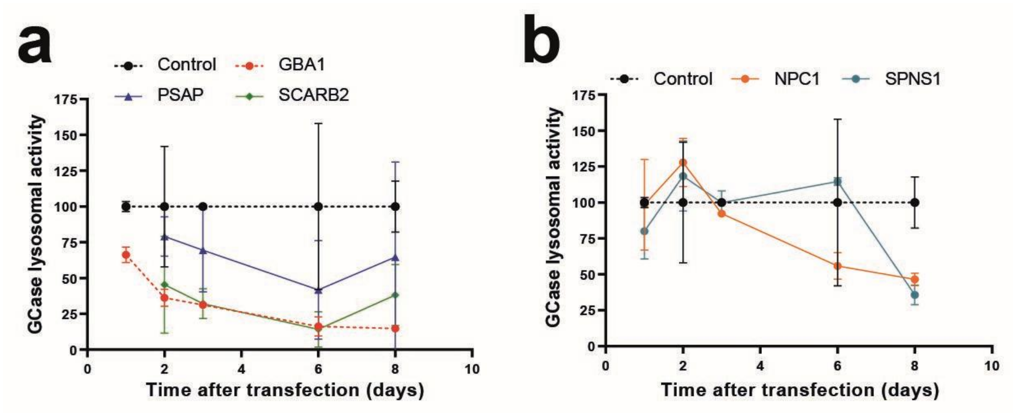

Figure S12 – Time-dependent GCase activity upon CRISPR KO: Variations of GCase lysosomal activity following CRISPR-Cas9 KO of control genes (GBA1, PSAP, and SCARB2) and identified modulators NPC1 and SPNS1 up to 8 days after transfection with the corresponding sgRNA.

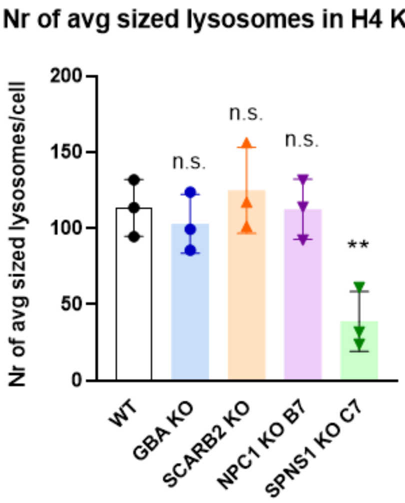

Figure S13 – Lysosomes phenotypes in KO cell lines: Co-staining between GCase and LAMP1 in Wild Type, GBA1 KO, SCARB2 KO, NPC1 KO and SPNS1 KO H4 cells. The number of lysosomes with an average size (based on Wild Type controls) was determined by fluorescence microscopy.

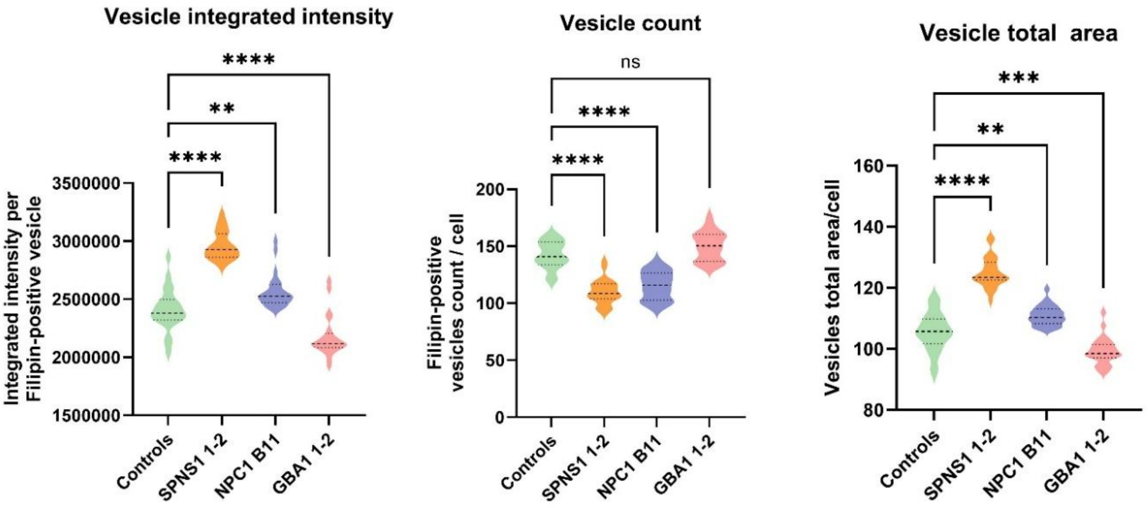

Figure S14 – Filipin staining in WT, GBA1 KO, NPC1 KO, and SPNS1 KO H4 cells: After staining with filipin, cells were imaged and analysis of fluorescence allowed to retrieve the area (left panel) and the integrated fluorescence intensity (right panel) of filipin-positive vesicles.

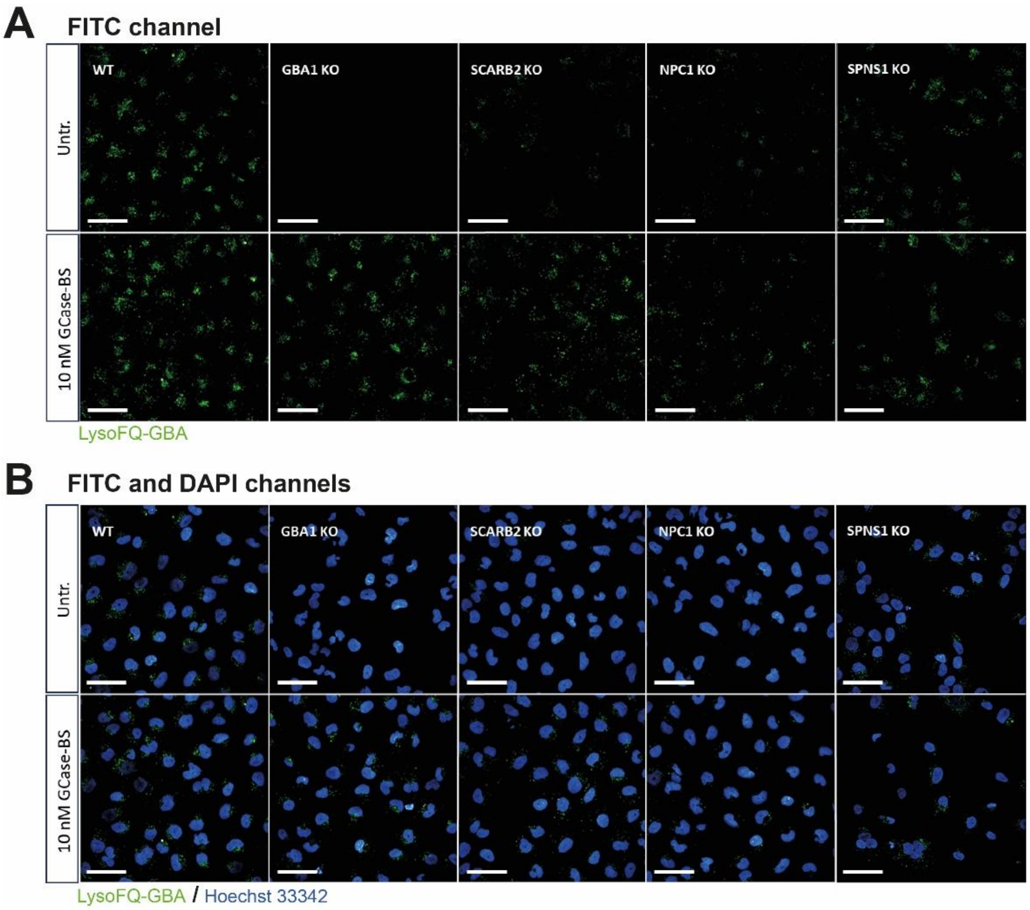

Figure S15. Lysosomal GCase activity upon treatment with Brainshuttle™ GCase. Representative images underlying the quantification of LysoFQ-GBA signal in H4 WT, GBA1, SCARB2, and SPNS1 KO cells upon 2 h treatment with 10 nM Brainshuttle™ GCase. (A) FITC channel, (B) Merge of FITC and DAPI channels. Scale bars: 100 µm.

Supplementary References

1. Deen, M. C. *et al.* A versatile fluorescence-quenched substrate for quantitative measurement of glucocerebrosidase activity within live cells. *Proc. Natl. Acad. Sci.* **119**, e2200553119 (2022).

2. Piccioni, F., Younger, S. T. & Root, D. E. Pooled Lentiviral-Delivery Genetic Screens: Pooled Lentiviral-Delivery Genetic Screens. in *Current Protocols in Molecular Biology* (eds. Ausubel, F. M. et al.) 32.1.1-32.1.21 (John Wiley & Sons, Inc., Hoboken, NJ, USA, 2018). doi:10.1002/cpmb.52.

3. De Leeuw, C. A., Mooij, J. M., Heskes, T. & Posthuma, D. MAGMA: Generalized Gene-Set Analysis of GWAS Data. *PLOS Comput. Biol.* **11**, e1004219 (2015).

4. Trubetskoy, V. *et al.* Mapping genomic loci implicates genes and synaptic biology in schizophrenia. *Nature* **604**, 502–508 (2022).

5. Giambartolomei, C. *et al.* Bayesian Test for Colocalisation between Pairs of Genetic Association Studies Using Summary Statistics. *PLoS Genet.* **10**, e1004383 (2014).

6. Lopes, K. D. P. *et al.* Genetic analysis of the human microglial transcriptome across brain regions, aging and disease pathologies. *Nat. Genet.* **54**, 4–17 (2022).

7. Kosoy, R. *et al.* Genetics of the human microglia regulome refines Alzheimer's disease risk loci. *Nat. Genet.* **54**, 1145–1154 (2022).

8. Nalls, M. A. *et al.* Identification of novel risk loci, causal insights, and heritable risk for Parkinson's disease: a meta-analysis of genome-wide association studies. *Lancet Neurol.* **18**, 1091–1102 (2019).

9. Jong, T., Gehrlein, A., Sidransky, E., Jagasia, R. & Chen, Y. Characterization of Novel Human β-glucocerebrosidase Antibodies for Parkinson's Disease Research. *J. Park. Dis.* **14**, 65–78 (2024).

10. Hu, M. *et al.* Parkinson's disease-risk protein TMEM175 is a proton-activated proton channel in lysosomes. *Cell* **185**, 2292-2308.e20 (2022).

Contributions

The 23andMe research team provided GWAS summary statistics for Parkinson's disease in the 23andMe cohort.

Data availability

The full Parkinson's disease summary statistics from 23andMe can be obtained under an agreement that protects the privacy of 23andMe research participants (https://research.23andme.com/collaborate/#publication).
