# Supplementary material for: Enzyme Activity-Based Genome-wide Screening for Modifiers of Lysosomal Glucocerebrosidase Uncovers Candidate Risk Factors for Parkinson’s Disease
Source: ACS Cent Sci. 2025 Sep 3;11(10):1933–45. doi: 10.1021/acscentsci.5c00240 (PMC12550621; doi:10.1021/acscentsci.5c00240)
Supplement: Supplementary file 2 [file oc5c00240_si_002.pdf]

oc-2025-00240j.R1

Name: Peer Review Information for "Enzyme activity-based genome-wide screening for modifiers of lysosomal glucocerebrosidase uncovers candidate risk factors for Parkinson disease"

First Round of Reviewer Comments

Reviewer: 1

Comments to the Author

In this manuscript the authors identify a set of genetic modifiers of glucocerebrosidase (GCase) activity, a subject of much interest in the Parkinson's disease field. This work compliments two recently published papers-one which used an arrayed CRISPR activation screen, and the other a CRISPR interference screen, to nominate genes that modify GCase activity. Since these three screens differ in the cell types used, mode of genetic perturbation, and GCase activity reporter, they provide distinct and only partially overlapping information. The set of genes identified in this manuscript will be a useful resource for the field and can be viewed as a bonafide set of modifiers due to the extensive optimization of their screen and subsequent secondary screening work. This work represents an advancement in understanding the penetrance of GBA-PD. Still, the manuscript would benefit from minor modifications outlined below.

-From the methods section it is unclear how the three rounds of enrichment played into calling hits. Were the fold changes in sgRNA abundance relative to non-tranduced from the three rounds averaged, or was the most enriched round weighted differently?

-In Figure S9, please provide molecular weight markers on the GCase and NPC1 blots which would be informative given the presence of multiple bands

-Throughout the manuscript there is no mention of statistics used in the figures. This is notably absent in figure 4. Please provide information on what each data point represents (i.e each point on the column plot represents a median fluorescence intensity for an experimental replicate), statistical test used to determine significance, and what each * above the column plots represents.

-Many microscopy images contain scale bars but there is no mention in the figure legend what distance the bars equal. Other images do not contain scale bars at all. Each image should have a scale bar and info in the corresponding figure legend.

-In Figure 4G/H the authors report a lack of colocalization between GCase and LAMP2 in each KO line. However, this appears to be driven by having lower GCase in the lines. In the NPC1 KO line it appears that the little GCase present colocalizes with LAMP2. It would be helpful to show a low exposure and high exposure image for GCase and potentially rerun the colocalization analysis with thresholding that permits the inclusion of lower intensity GCase signal in the colocalization with LAMP2.

-In the analysis in Table 1 the authors are making the case that the inclusion of SPNS1 and SPTTB to an existing set of lysosomal genes increases polygenic risk burden associated with lewy body pathology and progression to dementia. Are SPNS1 and SPTTB the only two additional lysosomal genes that appeared as CRISPR screen hits? If not, what was the criteria for their inclusion and the exclusion of other genes?

-The authors should show images of lysoFQ following addition of Brain Shuttle GCase. There should also be more of an explanation of this fusion protein. The fact that it rescues GCase activity in SCARB2 KO cells is surprising given that it would not be expected to be trafficked effectively to the lysosome in these cells. Rather is this protein being taken up from the extracellular space? Please provide some additional context and information in the methods section regarding this experiment.

-In the discussion section the authors write "Our experiments with substrates for other lysosomal enzymes, GalA and Cathepsin B, indicate that such perturbations are, to a considerable extent, specific to GCase activity." However, in SPNS1 and NPC1 KO cells

there is 3-4x increase in GalA enzymatic activity. While this isn't a reduction in activity it's a notable phenotype. The sentence should be either removed or revised and the authors are encouraged to discuss the pronounced effect SPNS1 and NPC1 KO have on GalA activity.

Reviewer: 2

Comments to the Author

The manuscript (oc-2025-00240j), titled Enzyme activity-based genome-wide screening for modifiers of lysosomal glucocerebrosidase uncovers candidate risk factors for Parkinson disease, touches upon GBA-PD in which mutations in GBA1 encoding GCase are associated with Parkinson Disease (PD).

The study aims to identify genetic modifiers of GCase function that influence the occurrence of GBA-PD. For this, a live cell GCase activity-based CRISPR-platform was employed to perform genome-wide screening for novel regulators of lysosomal GCase activity.

Reported screening hits are genes linked by GWAS studies to development and progression of PD. This can be viewed as validation of the approach. In addition, two lysosomal transporter genes encoding the lysosphospholipid transporter SPNS1 and the cholesterol transporter NPC1 were among the hits. In the case of SPNS1 an allele was identified is associated with increased risk of PD. Disruption of SPNS1 was confirmed to impair lysosomes, as earlier noted. Compromised GCase activity NPC patients has earlier been observed. The findings regarding SPNS1 are entirely novel and will attract considerable attention.

The authors conclude that "dysfunction of many PD associated genes converge to impact lysosomal GCase activity and thereby contribute to disease pathogenesis."

The investigation was conducted with true state-of-the-art techniques and methods. The study is well introduced, and the obtained data are presented in a clear manner. The authors are applauded with the investigation and the quality of the presented manuscript.

Specific comments.

Comment 1.

There is ample literature on SPNS1, the lysosomal transporter. Some of the papers link SPNS1 to general lysosomal dysfunction, while other suggest specific "substrates for transport" by SPNS1 the authors might spend more attention in the discussion to this literature on SPNS1.

Is specific accumulation of lysophospholipids, or even lysosphingolipid, impacting GCase activity or is it rather the general lysosomal dysfunction associated with defective SPNS1?

Comment 2.

The authors might explain why no other genes impacting the lysosomal milieu, and hence GCase activity, were no prominent screening hits. For example, genes impacting on lysosomal proton-pump activity and thus lysosomal pH: GCase is a known to be a very pH-sensitive enzyme.

Comment 3.

The manuscript provides a compelling case that lysosomal GCase activity may be negatively influenced by the presence of some known PD associated gene abnormalities. On the other hand, studies from the Gaucher field do not suggest unequivocally that the residual activity of specific GCase mutations influences the risk for Parkinson disease. The authors might consider addressing this conundrum. Taken together the above, an explanation could be that a defective GCase is not sufficient to increase the risk for PD but that accompanying additional genetic or even non-genetic modifiers must be present for an increased risk. The opinion of the authors regarding this thought is appreciated.

Note.

Introduction (lines 57-59): "LIMP-2 is itself modified by mannose-6-phosphate in the Golgi apparatus, and is recognized by the mannose-6-phosphate (M6P) receptor (MPR) and the formation of a ternary complex of these proteins enables trafficking of GCase to lysosomes. Ref 8."

This view might not be shared by the Saftig group in Kiel. There, LIMP-2 (encoded by the SCARB2 gene) was firstly identified and, in a collaboration with Genzyme researchers, the

role of LIMP-2 in sorting newly made GCase to lysosomes was demonstrated (Reczek et al., listed as ref 7). The Kiel researchers advocated rather a M6P receptor independent routing of LIMP-2/GCase to lysosomes, (Saftig P, Klumperman J. Lysosome biogenesis and lysosomal membrane proteins: trafficking meets function. Nat Rev Mol Cell Biol. 2009 Sep;10(9):623-35), consistent with the findings made with I-cell patient fibroblasts of normal GCase levels, sharply contrasting with reduced levels of other lysosomal hydrolases dependent on M6PR for routing to lysosomes.

Author's Response to Peer Review Comments:

Dear Editor,

Thank you for the positive news regarding our Manuscript (ID: oc-2025-00240j) titled "Enzyme activity-based genome-wide screening for modifiers of lysosomal glucocerebrosidase uncovers candidate risk factors for Parkinson disease" and for the interest of the journal. We appreciate your time in handling this manuscript and have worked to address all the points raised by the reviewers. We believe the resulting manuscript is significantly improved. A detailed set of responses is attached as a .pdf file and a version of the manuscript with relevant changes highlighted.

Sincerely,

David

We thank the editor and reviewers for their time spent in handling and assessing our manuscript. We appreciate the interest in our research and we thank the reviewers for their feedback. We have prepare a comprehensive reply to the comments from the reviewers that includes updates to the manuscript and figures with all text updates highlighted within the manuscript in yellow. Overall, we believe that the resulting manuscript is significantly improved and expect that the work will prove of interest to various communities.

Editor's Comments:

We have edited and updated the manuscript to fulfil all the formatting requests. Please find below a list detailing all the modifications that have been made to that effect.

1. Please place an asterisk (*) after the corresponding author name(s) in the author list.

Author list has been edited both in the manuscript and in the SI file.

2. Graphics: If a figure has parts labeled (i.e. a, b, etc.), each part must be mentioned in the figure caption. Figure 4j is currently not mentioned in the caption.

A description of Figure 4j has been added to the caption of figure 4.

3. Supporting Information: If the manuscript is accompanied by any Supporting Information for Publication, a brief description of the supplementary material is required in the manuscript, before the reference list. The appropriate format is: Supporting Information. Brief statement in non-sentence format listing the contents of the material supplied as Supporting Information. Please list each supporting item individually.

A brief description of the content of Supporting Information has been added in a short section before the reference list.

4. Supporting Information: Please label all graphics/tables in the following format: “Figure S1, S2…”, “Scheme S1, S2….” or “Table S1, S2…”, etc.

All the elements in the SI have been labelled according to the requested format.

5. Supporting Information: Please number all pages in the following format: S1, S2, S3,

   etc Pages in the SI have been numbered as requested.

6. Supporting Information: Please remove the line-numbering from your Supporting Information file.

We have removed the line-numbering from the SI file.

7. Synopsis: ACS Central Science requires a brief synopsis. The synopsis should be no more than 200 characters (including spaces) and should reasonably correlate with the Table of Contents (TOC) graphic. The synopsis is intended to explain the importance of the article to a broader readership across the sciences. Please place your synopsis in the manuscript file after the TOC graphic and label as “Synopsis.”

The following synopsis has been added at the end of the manuscript:

A fluorogenic substrate of GCase enables genome wide screening for genes than influence its activity and reveals candidate risk factors for PD – showcasing the power of activity-based screening.

Reviewers Comments:

Reviewer 1

Comment 1: From the methods section it is unclear how the three rounds of enrichment played into calling hits. Were the fold changes in sgRNA abundance relative to non-tranduced from the three rounds averaged, or was the most enriched round weighted differently?

Reply 1: Reviewer 1 brings up a good point. We found that the additional rounds of enrichment did not significantly improve the data quality and statistics. We have added a sentence in the manuscript to report this point as well as adding an additional figure in the SI (Figure S7). For data analysis we ended up using the most enriched round for both screens (round 2 for the pilot, round 3 for the genome-wide screen). Please note that each round was performed from 3 independent cell populations which were independently transduced, expanded, and sorted. Results obtained from the 3 populations were averaged to lead to the final list of hits.

Comment 2: In Figure S9, please provide molecular weight markers on the GCase and NPC1 blots which would be informative given the presence of multiple bands

Reply 2: The missing molecular weight markers in figure S9 result from an oversight. We thank the reviewer for pointing it out and we revised the figure accordingly.

Comment 3: Throughout the manuscript there is no mention of statistics used in the figures. This is notably absent in figure 4. Please provide information on what each data point represents (i.e each point on the column plot represents a median fluorescence intensity for an experimental replicate), statistical test used to determine significance, and what each * above the column plots represents.

Reply 3: The details requested have been added where applicable, *i.e.* within the legends of figures 2 and 4.

Comment 4: Many microscopy images contain scale bars but there is no mention in the figure legend what distance the bars equal. Other images do not contain scale bars at all. Each image should have a scale bar and info in the corresponding figure legend.

Reply 4: The microscopy images as well as the corresponding length they represent have been added for all the images in the manuscript (panels 1C, 4C, and 4G) and the corresponding figures legends have been updated accordingly.

Comment 5: In Figure 4G/H the authors report a lack of colocalization between GCase and LAMP2 in each KO line. However, this appears to be driven by having lower GCase in the lines. In the NPC1 KO line it appears that the little GCase present colocalizes with LAMP2. It would be helpful to show a low exposure and high exposure image for GCase and

potentially rerun the colocalization analysis with thresholding that permits the inclusion of lower intensity GCase signal in the colocalization with LAMP2.

Reply 5: We thank the reviewer for pointing out this issue and, to address this point, we have normalized the number of GCase spots colocalizing with LAMP1 for the total number of GCase spots (*ie* the amount of folded GCase per cell). When normalized to the amount of folded GCase, we can still observe a trend towards a lower GCase colocalization with LAMP1 compared to the wild type cells but not the significant decrease previously reported. We have updated figure 4h to reflect this new normalization, as well as edited the text in the manuscript as follows:

"Examining WT, *GBA1*, *SCARB2*, *NPC1*, and *SPNS1* KO cell lines, using LAMP1 as a lysosomal marker, when we accounted for the decreased levels of folded GCase seen in the knock-out lines, we observed a trend towards a lower extent of colocalization between GCase and LAMP1 in the *GBA1*, *SCARB2*, and *NPC1* cell lines (Figure 4g,h), which was generally consistent with our immunoblot results (Figure 4d,e). In the *SPNS1* KO cells, however, we observed a decrease in the amount of mature folded lysosomal GCase (Figure 4h) but immunoblot data showed no major change in overall GCase protein levels (Figure 4d,e)."

Comment 6: In the analysis in Table 1 the authors are making the case that the inclusion of SPNS1 and SPTTB to an existing set of lysosomal genes increases polygenic risk burden associated with lewy body pathology and progression to dementia. Are SPNS1 and SPTTB the only two additional lysosomal genes that appeared as CRISPR screen hits? If not, what was the criteria for their inclusion and the exclusion of other genes?

Reply 6: To clarify, there are quite a number of significant hits in the CRISPR screen but of these SPNS1 and SPTTB are the only *additional genes* located in proximity to *significant GWAS SNPs* that have not already been included in the curated list of lysosomal genes from the Molecular Signatures database used in our previously published lysosomal PD-PRS. To clarify the text we made some modification to the language:

"At the genome-wide significance threshold of p<5e-8, the revised lysosomal PRS included two additional loci that have not been previously included, which are proximal to the *SPNS1* and *SPTSSB* genes."

Comment 7: The authors should show images of lysoFQ following addition of Brain Shuttle GCase. There should also be more of an explanation of this fusion protein. The fact that it rescues GCase activity in SCARB2 KO cells is surprising given that it would not be expected to be trafficked effectively to the lysosome in these cells. Rather is this protein being taken up from the extracellular space? Please provide some additional context and information in the methods section regarding this experiment.

Reply 7: Reviewer 1 raises a good question here. To clarify, however, we previously showed with previous work that the Brainshuttle reaches the lysosomes by endocytosis after binding to the transferring receptors. Therefore, LIMP2-associated trafficking is bypassed. We have revised this section of the manuscript to make this more clear:

"Notably, because the BrainShuttle GCase can reach lysosomes by endocytosis after binding to the transferrin receptor it bypasses the need to bind to LIMP2.[54]"

We have added representative images of LysoFQ-GBA following treatment with the BrainShuttle GCase in the Supplementary Information (new Figure S15).

Comment 8: In the discussion section the authors write "Our experiments with substrates for other lysosomal enzymes, GalA and Cathepsin B, indicate that such perturbations are, to a considerable extent, specific to GCase activity." However, in SPNS1 and NPC1 KO cells there is 3-4x increase in GalA enzymatic activity. While this isn't a reduction in activity it's a notable phenotype. The sentence should be either removed or revised and the authors are encouraged to discuss the pronounced effect SPNS1 and NPC1 KO have on GalA activity.

Reply 8: While the increase of GalA activity is indeed a notable phenotype, it does not cause a loss of function as what is observed with GCase. The relationship between SPNS1 or NPC1 knockout and GalA activity are not central to the manuscript, and we note that we do not have adequate data to discuss possible causes. However, we appreciate the reviewer's point and have noted this in the discussion and revised the paragraph in the main text to highlight this observation and offer some reasoned speculation:

"Our experiments with substrates for other lysosomal enzymes indicate that such perturbations are generally not deleterious for the function of lysosomal enzymes other than GCase. Indeed, changes arising from KO of *NPC1* or *SPNS1* do not affect Cathepsin B and, surprisingly, increase the activity of GalA. These observations underscore the sensitivity of lysosomal GCase and suggest why mutations in *GBA1* might be commonly linked to PD yet exhibit relatively low penetrance. We speculate that GCase is sensitive to genetic perturbations and environmental stimuli that influence the lysosomal environment and that such sensitivity can be compounded by even relatively mild heterozygous variants in *GBA1*."

Reviewer 2

Comment 1: There is ample literature on SPNS1, the lysosomal transporter. Some of the papers link SPNS1 to general lysosomal dysfunction, while other suggest specific "substrates for transport" by SPNS1 the authors might spend more attention in the discussion to this literature on SPNS1. Is specific accumulation of lysophospholipids, or

even lysosphingolipid, impacting GCase activity or is it rather the general lysosomal dysfunction associated with defective SPNS1?

Reply 1: This is an interesting question. We have revised the text within the discussion to note these possibilities and direct attention to the need for more detailed mechanistic studies to dissect this question. The text has been updated as follows:

"Moreover, though we only observed a slight increase in GlcSph levels in *SPNS1* KO cells, it is notable that, in recently described *SPNS1* KO mice, there are clear increases in sphingosine levels, including both GCase substrates GlcSph and GlcCer, and emergence of a LSD phenotype – pointing to the development of clear lysosomal dysfunction.[57] While tempting to speculate that accumulation of the SPNS1 substrates,[42,43] LPC and LPE, within lysosomes may themselves impair GCase function, it is notable that we see a decrease in the levels of folded lysosomal GCase in *SPNS1* KO cells, which perhaps suggests that loss of SPNS1 induces a defect in the trafficking of GCase to lysosomes. Resolution of this question will require more detailed mechanistic cellular studies.[7]"

Comment 2: The authors might explain why no other genes impacting the lysosomal milieu, and hence GCase activity, were no prominent screening hits. For example, genes impacting on lysosomal proton-pump activity and thus lysosomal pH: GCase is a known to be a very pH-sensitive enzyme.

Reply 2: Yes, thank you, we do indeed see several such genes and have clarified this item within the text. In particular, we now underscored this observation in the main text and the discussion:

Main Text: "From among our observations (Supporting Table S2) there are several that are directly linked to lysosomal homeostasis. Indeed, we also observed 12 genes encoding subunits of the Vacuolar-type ATPase (V-ATPase) (*ATP6V0D1, ARP6V1C1, ATP6V1H*, *ATP6V1D*, *ATP6V1G1, ATP6AP1, ATP6V0C, ATP6V1F, ATP6V1A, ATP6V1E1, ATP6V0B, ATP6V1B2*), of which *ATP6V0C*, *ATP6V1A*, and
ATP6V0A1 are linked to neurodevelopmental disorders,[28] underscoring the importance of the VATPase in maintaining the acidic environment of lysosomes coupled with the known pH-sensitivity of GCase.[29]"

Discussion: "While the number of genes observed precludes detailed discussion, perusal reveals many genes that have logical links to lysosomal function and GCase activity. In particular, as noted above, we found that many genes implicated in lysosomal maturation, lysosomal pH homeostasis, and lysosomal lipid composition influence GCase activity."

Comment 3: The manuscript provides a compelling case that lysosomal GCase activity may be negatively influenced by the presence of some known PD associated gene abnormalities. On the other hand, studies from the Gaucher field do not suggest unequivocally that the residual activity of specific GCase mutations influences the risk for Parkinson disease. The authors might consider addressing this conundrum. Taken together the above, an explanation could be that a defective GCase is not sufficient to increase the risk for PD but that accompanying additional genetic or even non-genetic modifiers must be present for an increased risk. The opinion of the authors regarding this thought is appreciated.

Reply 3: This is an important question raised by the reviewer. We have added some mention of this issue to the discussion:

"This suggests that upstream perturbations – both genetic and environmental – may converge on GCase to impair its activity. Interestingly, the penetrance of PD among carriers of *GBA1* mutations is modest, suggesting that these mutations alone may not be sufficient to cause PD. This suggests that additional genetic impairments may be implicated in triggering the development of *GBA1*-associated PD."

Note: Introduction (lines 57-59): "LIMP-2 is itself modified by mannose-6-phosphate in the Golgi apparatus, and is recognized by the mannose-6-phosphate (M6P) receptor (MPR) and the formation of a ternary complex of these proteins enables trafficking of GCase to lysosomes. Ref 8." This view might not be shared by the Saftig group in Kiel. There, LIMP-2 (encoded by the SCARB2 gene) was firstly identified and, in a collaboration with Genzyme researchers, the role of LIMP-2 in sorting newly made GCase to lysosomes was demonstrated (Reczek et al., listed as ref 7). The Kiel researchers advocated rather a M6P receptor independent routing of LIMP-2/GCase to lysosomes, (Saftig P, Klumperman J. Lysosome biogenesis and lysosomal membrane proteins: trafficking meets function. Nat Rev Mol Cell Biol. 2009 Sep;10(9):623-35), consistent with the findings made with I-cell patient fibroblasts of normal GCase levels, sharply contrasting with reduced levels of other lysosomal hydrolases dependent on M6PR for routing to lysosomes.

Reply to the note:

We thank the reviewer for noting this point. We have added text to two different locations in the manuscript to clarify this important point.

Main text: "However, it is important to note that an alternative proposal is that the LIMP-2-GCase complex traffics to lysosomes in a M6P-indepedent manner.[7]"

Discussion: "Interestingly, we also observed *GNPTAB*, which is essential for Man6P-dependent trafficking, which might suggest a direct requirement for Man6P for trafficking of

GCase. However, we note that even in the *SCARB2* KO cells, though at lower levels, we still observe folded lysosomal GCase, which supports the proposal that GCase can indeed reach the lysosome – at least in part – through a Man6P-independent process.[7]”
